# Genome-Based Taxonomy of the Genus *Stutzerimonas* and Proposal of *S. frequens* sp. nov. and *S. degradans* sp. nov. and Emended Descriptions of *S. perfectomarina* and *S. chloritidismutans*

**DOI:** 10.3390/microorganisms10071363

**Published:** 2022-07-06

**Authors:** Margarita Gomila, Magdalena Mulet, Elena García-Valdés, Jorge Lalucat

**Affiliations:** 1 Microbiology (Biology Department), Universitat de les Illes Balears, 07122 Palma de Mallorca, Spain; marga.gomila@uib.es (M.G.); mmagdalena.mulet@uib.es (M.M.); elena.garciavaldes@uib.es (E.G.-V.); 2 Institut Mediterrani d’Estudis Avançats (IMEDEA CSIC-UIB), 07190 Mallorca, Spain

**Keywords:** *Stutzerimonas*, phylogenomic species, core-genome phylogeny, *S. perfectomarina*, *S. chloritidismutans*, *S. frequens*, *S. degradans*

## Abstract

*Stutzerimonas* is a recently proposed genus within the *Pseudomonadaceae* comprising strains in the formerly phylogenetic group of *Pseudomonas stutzeri*. At least sixteen named species have to be included in the genus, together with 22 genomovars of *Stutzerimonas stutzeri*. To clarify the taxonomy of *Stutzerimonas*, a core-genome phylogeny of 200 strains in the genus was inferred and monophyletic strains with average nucleotide identities (ANIb) with values equal to or higher than 95 were grouped in the same phylogenomic species. A total of 45 phylogenomic species within the genus *Stutzerimonas* were detected in the present study. Sixteen phylogenomic species correspond to already named species, although three of them are not yet validated and two are proposed in the present study. A synonymy was detected between *P. kunmingensis* and *S. chloritidismutans*, both members of phylogenomic species 3, with a prevalence of the *S. chloritidismutans* name. The correspondence of the phylogenomic species to the genome taxonomy database classification (GTDB taxonomy) is discussed. Combining phylogenomic and phenotypic data, two novel species are described (*Stutzerimonas frequens* and *Stutzerimonas degradans*) and two species descriptions are emended (*Stutzerimonas perfectomarina* and *Stutzerimonas chloritidismutans*).

## 1. Introduction

The genus *Stutzerimonas* has been recently proposed within the *Pseudomonadaceae* family and includes species formerly ascribed to the genus *Pseudomonas* in the so-called *Pseudomonas stutzeri* phylogenetic group, also known as *P. stutzeri* complex. This group contains currently 16 named species, three of them with not yet validated names [1] (Table 1 and Supplementary Table S1). The genus proposal was sustained by phylogenomic analysis based on the core-genome phylogeny of the *Pseudomonadaceae* and by several indices based on the genes shared by the species members of the new genus, but also by their phenotypic characteristics, including denitrification properties and the absence of arginine dihydrolase activity [2]. Some strains are able to fix nitrogen, a characteristic not present in the other *Pseudomonas* phylogenetic groups. *Stutzerimonas stutzeri* is the type species of the genus and is characterized by its subdivision into 22 different genomic groups that can be differentiated by experimental DNA-DNA hybridizations and by genomic indices with values below the species threshold, but that cannot be differentiated phenotypically. The term genomovar (gv) was coined to design provisionally these genotypically similar groupings [3,4]. A reference strain was proposed for each genomovar. Some of the genomovars are coincident with species later described in the group.

Former *Pseudomonas* species transferred to the new genus *Stutzerimonas* are: *Stutzerimonas stutzeri* (gv1), *Stutzerimonas azotifigens*, *Stutzerimonas balearica* (gv6), *Stutzerimonas chloritidismutans* (gv3), *Stutzerimonas kirkiae*, *Stutzerimonas kunmingensis* (gv3), *Stutzerimonas nosocomialis*, *Stutzerinomas urumqiensis*, *Stutzerimonas xanthomarina*, and *Stutzerimonas zhaodongensis* (gv20) [2]. The corresponding genomovar is indicated within parenthesis after the species name. Most of these names have been proposed and are listed in Validation List 204 published in the International Journal of Systematic and Evolutionary Microbiology [5]. Other recognized species in the same phylogenetic group are *Pseudomonas nitrititolerans* (gv8), *Pseudomonas perfectomarina* (gv2), and *P. tarimensis*, as well as three other published species whose names have not been validated yet: “*Pseudomonas lopnurensis*”, “*Pseudomonas saudiphocaensis*”, and “*P. songnenensis*”.

The Genome Taxonomy Data Base (GTDB) [6] relies on the relative evolutionary distance (RED) that is calculated by means of the nucleotide sequences of 120 monocopy universal housekeeping genes and it is a well-accepted and comprehensive genome-based bacterial and archeal taxonomy. Besides other genera, the GTDB taxonomy release 06-RS202 (27 April 2021) differentiates within the *Pseudomonadaceae* four genera that include species in the genus *Stutzerimonas*: Pseudomonas_A with 46 species, Pseudomonas_N with 2 species, Pseudomonas_Q with 2 species, and Pseudomonas_R with 3 species. Nine of these species are represented solely by metagenome-assembled genomes (MAGs) as indicated in Figure S1.

The main aim of the present study is to clarify the taxonomy of species in the genus *Stutzerimonas*, assess the genomovar taxonomic status, and compare the current taxonomy with the proposed GTDB taxonomy. The phylogenomic analysis developed in the present study relies on the phylogeny of 200 strains derived from the concatenated sequences of the core-proteome of the genus and the species delimitation based on genomic indices (ANIb [7] and GGDC [8,9]). Each cluster of strains that are monophyletic and with genomic indices equal to or higher than 95% (ANIb) or 70% (GGDC) are considered phylogenomic species (pgs) in this research and follow the criteria proposed by Staley for a universal phylogenomic species concept [10]. In the present study, former genomovars (gv) of *P. stutzeri* are labeled pgs of *Stutzerimonas* maintaining the same number. *Stutzerimonas* named species are not given a pgs number when no correspondence can be detected with the previously described genomovars of *P. stutzeri*. To reach the mentioned objectives, genomes of 32 *Stutzerimonas* strains are sequenced and analyzed in this study together with genome sequences of related strains of publicly available databases in the NCBI (https://www.ncbi.nlm.nih.gov/ (accessed on 8 April 2022) and JGI (https://genome.jgi.doe.gov/portal/ (accessed on 8 April 2022)) websites.

Additionally, the reference strains of four phylogenomic species (2, 3, 5, and 7) together with other relevant strains available in our culture collection are characterized phenotypically. Combining new and previously published phenotypes, two genomovar reference strains are proposed as type strains of two new species in the genus *Stutzerimonas*, five species in the former *P. stutzeri* phylogenetic group are transferred to *Stutzerimonas,* and two species descriptions are emended. At least 30 other phylogenomic species are delineated within the genus *Stutzerimonas* and remain to be described as novel species when more strains and data become available in the future.

## 2. Materials and Methods

### 2.1. Bacterial Strains

The list of bacterial strain representatives of the phylogenomic species analyzed in the present study is given in Table 1. All analyzed strains, together with their genome sequence accession numbers, habitat, and geographical origin are given in Table S1.

### 2.2. Genome Sequencing

Genomic DNA was isolated from 32 strains formerly classified in the *P. stutzeri* phylogenetic group using the Wizard Genomic DNA Purification kit (Promega, Madison, WI, USA) according to the manufacturer’s instructions. The list of genome strains obtained in this study is indicated in Table S1. Genomes were sequenced at different companies, i.e., Macrogen, BGI, or Lifesequencing, using different pair-end reads sequencing (100 bp, 150 bp, and 250 pb) depending on the company. The obtained Illumina HiSeq 2000 paired-end library reads were assembled de novo using the Newbler Assembler version 2.9 program (Roche, Basel, Switzerland). The draft genome was annotated using the NCBI Prokaryotic Genome Annotation Pipeline. The Whole Genome Shot-gun projects for all strains sequenced have been deposited in DDBJ/ENA/GenBank. Detailed accession numbers are indicated in Table S1. The G + C content was calculated directly from the genome sequences and is shown in Table S1. The genome sizes and the number of contigs are indicated in Table S1.

### 2.3. Phylogeny and Species Genomic Indices

A core-proteome analysis was performed for all strains given in Table S1 using the M1CR0B1AL1Z3R web server (https://microbializer.tau.ac.il/ (accessed on 1 May 2022); [11]). Briefly, the program extracted the protein-coding ORFs of the submitted genomes using Prodigal, and a homology search was conducted. Each ORF was queried against all other ORFs in the set of genomes, and orthologous genes were grouped by the best reciprocal hit. The minimal percent identities tested were set at 50, 60, 70%, and 80%, and the maximal permitted e-value was 0.01. A level of 70% was selected for the phylogenetic analyses as justified below. The phylogenetic trees were calculated by concatenating the sequences of the protein-coding genes present in all strains under analysis. Trees were constructed by maximum likelihood (ML). Bootstrap values of 100 replicates were calculated. The same analysis was also performed considering only the 200 *Stutzerimonas* genomes.

Average nucleotide identities were calculated by blast (ANIb) using the JSpecies web server (JSpecies.ribohost.com/ (accessed on 1 May 2022)) [7] and the Enveomics tool [12]. For the pairs of strains with ANIb values on the borderline for species differentiation, the whole genome relatedness was also calculated by the method described by Meier Kolthoff et al. [8,9] at the Genome to Genome Distance Calculator (GGDC) web server (https://www.dsmz.de/services/online-tools/genome-to-genome-distance-calculator-ggdc (accessed on 1 May 2022)).

A phyletic pattern of each strain was obtained at the M1CR0B1AL1Z3R web server (https://microbializer.tau.ac.il/ (accessed on 1 May 2022)). The presence or absence of 18,654 orthologous genes with 70% identity and a minimal e-value of 0.01 in the set of 200 *Stutzerimonas* genomes were recorded for each strain. The percentage of shared genes was calculated pairwise with the Jaccard index and represented in a dendrogram with the PAST program [13]. The Jaccard index of similarity was calculated as SJ = a/(a + b + c + d), in which a is the number of genes that were present in both genomes of each pair, b and c are the number of genes present in one strain but absent in the other, and d is the number of orthologs absent in both strains. The orthologous genes shared by *Stutzerimonas* strains were also represented in a splits tree decomposition as discussed by Huson and Bryant [14]. SplitsTree (version 5) is a software for computing unrooted phylogenetic networks from molecular sequence data.

### 2.4. Phenotypic Characterization

A total of 7 strains of the emended species descriptions or newly proposed species were characterized phenotypically and are listed in Table S2. It includes the pgs reference strains labeled as pgs2, pgs3, pgs5, and pgs7.

The morphological, physiological, and biochemical characteristics of the strains were determined using conventional microbiological tests following Cowan and Steel’s procedures [15], including the assessment of colony morphology, Gram staining, motility, oxidase and catalase activities, and growth on different media, and growth in the presence of NaCl (0–10% *w*/*v*) at different temperatures (4 °C, 6 °C, 10 °C, 22 °C, 30 °C, 37 °C, 42 °C, and 45 °C) and pH values (4, 5, 6, 7, 8, 9, 10 and 11). LB medium was used for routine cultivation. Production of pigments on King A and fluorescence on King B media was also tested as recommended by the manufacturer (Difco). Bacterial cell morphology was observed by electron microscopic examination of negatively-stained samples prepared as previously described [16]. Motility was determined by observation of cells in the early stationary growth phase under phase contrast light microscopy. The strains were also characterized by using Biolog GEN III commercial plates according to the manufacturer’s instructions. The plates were inoculated with cells previously cultured on LB medium. API20NE for strains ATCC 14405T and AW-1T were performed in our laboratory following the manufactures instructions. API 20NE test data for the other strains were retrieved from BacDive, a worldwide database for standardized bacterial information from the Leibniz Institute DSMZ (German collection of microorganisms and cell cultures (https://bacdive.dsmz.de/ (accessed on 8 April 2022)) or from previous publications as indicated in the references given in the corresponding descriptions.

Whole-cell fatty acid methyl ester (FAME), polyamines, and ubiquinone analyses were retrieved from previous data [17] for strains corresponding to pgs2, 3, 5, and 7 or from the web page of the CCUG (University of Gothenburg Culture collection: https://www.ccug.se/ (accessed on 8 April 2022)) as indicated in the respective species characterizations.

## 3. Results

### 3.1. Genome Characteristics of the New Sequenced Strains

The genome sizes for the 31 *Stutzerimonas* strains sequenced in this study ranged from 3,698,628 bp (strain V81) to 5,393,046 bp (strain A563/77) in the range described for the *Pseudomonadaceae*, and the GC content from 59.4% (*S. zhaodongensis* PE) to 65.2% (*Stutzerimonas* pgs17 strain 24A75) as shown in Table S1. The number of contigs ranged from 28 to 284 contigs. The GC values were in the range of the *Stutzerimonas* species [2] and the variation within a single pgs was not higher than 1%, as corresponds for strains of the same species. The genome of *P. kuykendallii* LMG 24364T was also sequenced because other studies included this species within the *P. stutzeri* phylogenetic group of species [18].

### 3.2. Core-Genome Phylogeny

**Figure 1:**
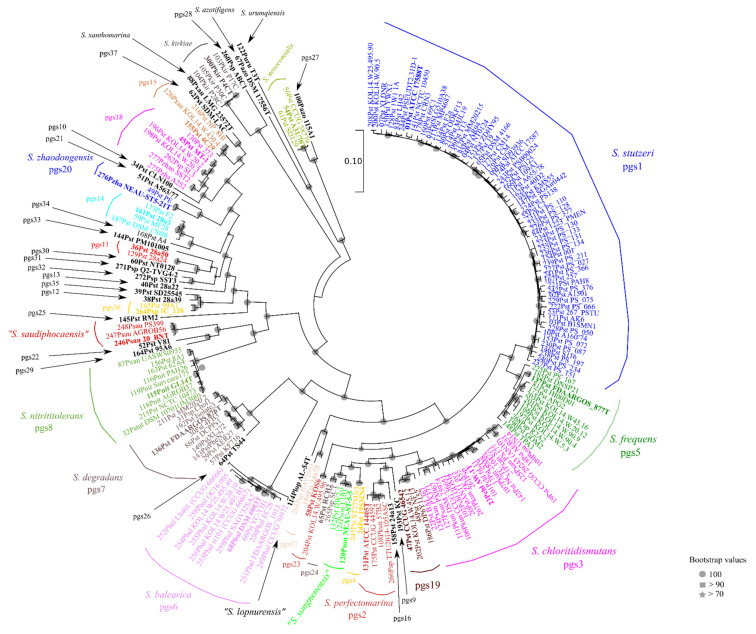
Core-genome phylogeny based on the concatenated sequences of 1054 core genes of 200 genomes of species in the *Stutzerimonas* genus. Tree was constructed using the maximum likelihood method. Bootstrap values of 100 replicates are indicated in the nodes with different symbols: circles indicate bootstrap values of 100, squares indicate values higher than 90%, and stars indicate values higher than 70%.

To infer the phylogeny of the genus *Stutzerimonas,* we included in the analysis as outgroup the genomes of the type strains of closely related genera, like *P. aeruginosa*, *Azotobacter,* and *Azomonas* species, all in the *Pseudomonadaceae* family. A phylogenetically more distant genus was not included because the number of orthologous genes diminished drastically. The analysis of the core-proteome was performed at 50, 60, 70%, and 80% identity and an e-value of 0.01. To reach a number of genes similar to other phylogenetic studies [19,20], a 70% identity was selected for further studies. A total of 22,914 orthologous genes were detected among 212 genomes studied when a minimal 70% identity was selected. Results presented in Figure S2 are based on the concatenated sequences of 666 core genes (a length of 243,405 aminoacids) and included as an outgroup of other species in the *Pseudomonadaceae*. Branches are supported with a high percentage of bootstrap values. All values are higher than 90% and most of them are 100%. *P. kuykendallii* was located outside the *Stutzerimonas* phylogenetic branch. Another phylogenetic analysis considered exclusively the 200 genomes of strains in the *Stutzerimonas* genus and is depicted in Figure 1. The core proteome was constituted by 1054 orthologs of the 18,654 orthologous genes found, with a length of 366,079 aminoacids, that is, approximately one-third of the genome length of the shortest *Stutzerimonas* genome: *S. saudiphocaensis* contains 3340 protein-coding sequences. The branching order and groupings in both phylogenetic trees, with and without outgroup, were almost identical. The branching order was identical for the 45 phylogenomic branches, with one exception: the branch of *S. azotifigens*/*S. urumqiensis* was separated earlier from the rest of the *Stutzerimonas* species when the outgroup was introduced in the analysis. No significant differences could be observed in the branching order within the 45 phylogenomic species defined in the present study. Concatenated core genome gene alignments are provided as supplementary information.

To complete the comparison of the phylogenomic approach of the present study with the GTDB taxonomy, an analysis was performed including the representative strains of all species defined in the GTDB taxonomy in which eleven species were represented by genome sequences derived from the analysis of metagenomes (MAGs). MAGs were not included in the core-phylogenetic analysis previously described in our study, due to discrepancies detected in the ANIb clustering, as explained below, and due to differences in the core proteome when the MAGs were included. The core proteome of the 62 genomes of pgs representative genomes depicted in Figure S3 is based on only 758 genes, a significantly lower number than the 1265 genes detected in the same analysis without including the MAGs. Interestingly, the analysis allowed us to identify the MAGs UBA7516 (Pseudomonas_A stutzeri_Q) as equivalent to *Stutzerimonas* pgs21, UBA2080 (Pseudomonas_A stutzeri_T) to *Stutzerimonas* pgs22, and CAL (Pseudomonas_A sp007713455) to *S. chloritidismutans*.

### 3.3. Digital Species Delineation (ANIb, GGDC, and Phyletic Pattern)

**Figure 2:**
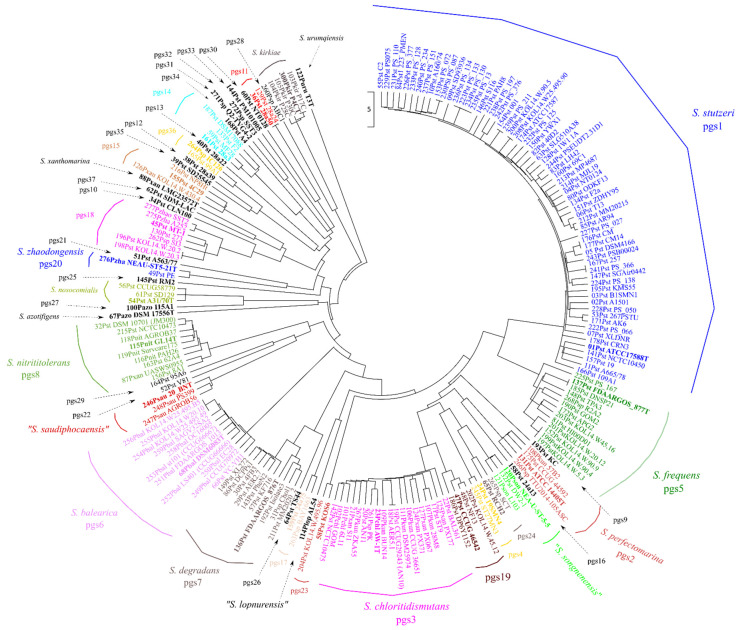
UPGMA dendrogram of the average nucleotide identities (ANIb) analysis of the 200 genomes of *Stutzerimonas* strains. Members of each phylogenomic species (pgs) are indicated with the same color.

A dendrogram was used to represent the ANIb matrix (26,450 pairwise comparisons) in Figure 2. All clusters equal to or above the 95% species threshold were monophyletic in the core-genome phylogeny, with the only exception of pgs7. The lowest value of the ANIb index among strains in pgs7 was 94.2, on the borderline for species circumscription, but the closest related strain was the representant of pgs26 at 92% (Table S3). The GGDC value was also calculated for these strains and the lowest value was 63.4%, also on the species borderline that was established at 70% similarity. The clear discontinuity of pgs7 strains from the other clusters allowed the delineation of a single pgs.

**Figure 3:**
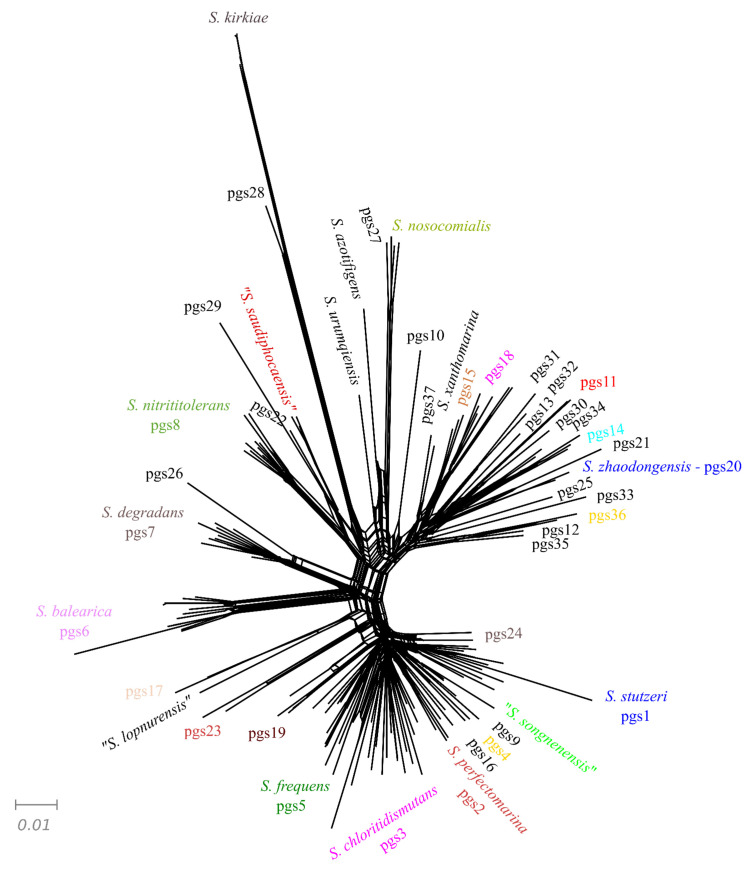
Splitstree representation of the phyletic pattern obtained in the analysis of the orthologous genes of 148 genomes selected from the 200 *Stutzerimonas* strains analyzed. Different pgs are indicated in different colors.

The clustering of strains obtained in the UPGMA dendrogram of the phyletic pattern (Jaccard index) was concordant with the core genome phylogeny and with the ANIb results. All strains of the same phylogenomic species were grouped in the same cluster with few exceptions: *S. songnenensis* and pgs24; pgs14 and pgs34 and the strains Px177 misclassified as *P. xanthomarina,* and; strain B1SMN1 included in the *S. stutzeri* group (Figure S4). The splits tree decomposition that was based for clarity on only 148 of the 200 genomes is shown in Figure 3, in which all strains of the same phylogenomic species were grouped. Representants of *S. stutzeri* pgs1 were selected randomly. These results are a good indication that strains in the same phylogenomic species are homogeneous in their gene content and that the genes shared among strains in the species and absent in the other close-related species are characteristic of the species.

Combining the core-genome phylogeny with the ANIb species circumscription, a total of 49 phylogenomic species within the genus *Stutzerimonas* were detected in the present study. Eight additional pgs were represented only by MAGs. Sixteen of the 49 pgs are already named species, although three of them are not yet validated. A synonymy was detected between *P. kunmingensis* and *S. chloritidismutans*, both members of pgs3, with a prevalence of the *S. chloritidismutans* name.

### 3.4. Phenotypic Characterization and Species Descriptions

Due to the incorporation of many strains to already named species, the descriptions of two of the validly named species have to be emended: *S. chloritidismutans* and *S. perfectomarina* (Table 2 and Table 3). The morphological, physiological, and biochemical characterizations of strains in both species are given in supplemental Table S3. Other well-studied strains were included in pgs5 and 7 and their phenotypic characteristics are given in the species descriptions and in supplemental Table S3. These characteristics were complemented with results of previous characterizations of *P. stutzeri* genomovars and are summarized in the species descriptions given in Table 4 and Table 5 for two proposed novel species: *Stutzerimonas frequens* (pgs5) and *Stutzerimonas degradans* (pgs7) as presented in the section of taxonomic conclusions.

## 4. Discussion

Taxonomic identification at the species level is a prerequisite for many microbiological studies. Table S1 summarizes the identification at the pgs level of the 200 genomes under study and it is remarkable that at least 66 of the 200 strains in the NCBI database are labeled as inconclusive in their taxonomic check (Table S1). Our results confirmed this low identification precision in a significant number of strains. For instance, genome sequences of strains KGS-2 and KGS-8 of *P. stutzeri* are excluded from reference sequences in the NCBI database because the genome length is too large. A detailed analysis in the present study demonstrated that the phylogenetic closely-related species type strain to both strains is *Pseudomonas reineckei* (90% of ANIb similarity) in the *Pseudomonas lutea* phylogenetic group, distant from *Stutzerimonas*.

A good concordance exists between the GTDB, the core-genome, and the 4-genes MLSA phylogenies as previously demonstrated for species in the *Pseudomonadaceae* [18]. The species in the genus *Stutzerimonas* of the present study are distributed in four possible monophyletic genera in the GTDB taxonomy: Pseudomonas_A, _Q, _N, and _R (Figure S1 and Figure 1) but the grouping in a single genus has been discussed in a previous publication [2]. The percentage of shared genes by strains in the *Stutzerimonas* genus justified the unification of the four GTDB genera in a single genus. The 200 strains analyzed in the core-genome phylogenetic tree of the genus *Stutzerimonas* are distributed in 49 phylogenomic species as assessed by means of genomic indices (ANIb and GGDC) and by the percentage of orthologous genes shared by strains of the same proposed phylogenetic species. The percentage of shared orthologous genes among strains of the same species is a measure of the genetic potential of this species and represents the genetic isolation from the rest of the strains under study.

A very good correlation was observed between the experimental DDH assessed by the percentage of hybridization or by the difference in the hybrid melting temperature (delta Tm) of previous studies in which the genomovars of *P. stutzeri* were described [21,22,23]. Both the ANIb and GGDC values confirmed the groupings of strains. In a recent study, Li and collaborators [19] have used a similar approach to study strains in the *P. stutzeri* complex, but with fewer genome sequences (123), and found 27 genomovars that are coincident with 27 phylogenomic species of the 49 described in the present study. The final assignation of the 200 strains is given in Table S1. It is worth mentioning that 22 pgs are singletons, represented by a single strain, which is an indication that many pgs remain still to be discovered.

Eleven of the 45 pgs proposed in this study are not present in the GTDB database (Table 1). Fifty-three species are delineated in the GTDB taxonomy, but 11 of the GTDB proposed species are represented only by MAGs and these were not considered in our phylogenetic analysis because the number of core genes diminished drastically when the MAGs were included. Moreover, inconsistencies were detected between the phylogenomic analysis and the genomic species indices when MAGs were included in the analysis. For instance, the MAG labeled UBA3235 (GCA_002362915) is classified in the GTDB taxonomy as a member of Pseudomonas_A stutzeri_L. Its closest relative in the core-genome analysis is the type strain of *S. lopnurensis* at a distance compatible with strains of the same pgs. However, the ANIb value was low (80%) and the MASH-ANI calculation at the autoMLST website indicated errors in the sequence. Several MAGS are labeled in the NCBI as “many fragments with little to no review of assembly other than reporting of standard assembly statistics”. On the other hand, the MAG labeled UBA2080 (GCA_002331645.1) was phylogenetically close to strain V81 (pgs22) and the ANIb value among both strains was 99%. Therefore, UBA2080 and V81, together with MAG NTP17 (GCA_015478845) have to be considered members of pgs22 (equivalent to Pseudomonas_A stutzeri_T). MAGs quality and completeness are probably the reasons for the discrepancies.

In the comparison between the pgs detected in this study and the GTDB taxonomy, we detected several inconsistencies. It is important to highlight that the genome sequences of the *S. stutzeri* type strains CGMCC 1.1803T and ATCC 17588T are located in two different species in the GTDB taxonomy, Pseudomonas_A stutzeri (pgs1) and Pseudomonas_A sp00320581.5 (pgs5), whereas the sequence of strain ATCC 17588T affiliates to pgs1 strains in the core-genome phylogeny with ANIb values in the species range with the rest of strains in pgs1. The inclusion of several MAG sequences in the GTDB taxonomy might be the reason for these inconsistencies.

Twenty strains of pgs3 are distributed in eight different species in the GTDB taxonomy: Pseudomonas_A chloritidismutans, Pseudomonas_A kunmingensis, Pseudomonas_A kunmingensis_A, Pseudomonas_A sp002692525, Pseudomonas_A sp. 007713455 (strain CAL), Pseudomonas_A stutzeri_AD (strain BAL361), Pseudomonas_A stutzeri_U (strains CCUG 29243 =AN10 and BUN14 are the only members), and Pseudomonas_A xanthomarina_A (strain PX_067). A relevant comment to these assignations is the case of strain ST-9 (pgs3) that is included in a different species, Pseudomonas_A sp002692525, together with 6 MAGs. In our opinion, the inclusion of MAGs with uncompleted genomes can deviate the ANIb analysis. However, our phylogenomic analysis demonstrates that all strains of pgs3 belong to the same phylogenomic species, for which the name *Stutzerimonas chloritidismutans* has been proposed and *S. kunmingensis* has to be considered a later synonym. The 20 strains of pgs 3 are monophyletic in the core-genome phylogeny and in the GTDB phylogeny and the lowest ANIb value among all strains was 96.4% (Table S3) confirming the correct affiliation to the same species.

*P. xanthomarina* strains are distributed into two species in the GTDB taxonomy: Pseudomonas_A xanthomarina and Pseudomonas_A xanthomarina_A. Surprisingly, the type strain of *P. xanthomarina* is represented three times and in both species. This anomaly can be attributed also to the inclusion of 15 MAGs in the analysis. Another relevant discrepancy is that seven strains are classified within *P. xanthomarina* in the NCBI database, but only the type strain belongs to this species; the six other strains are distributed in four different pgs (Table S1).

The species in the former *P. stutzeri* phylogenetic group (*P. stutzeri* species complex) are characterized by a high phenotypic and genotypic diversity [21]. Strains identified as *P. stutzeri* were classified in 22 genomic groups (genomovar, gv) that were not considered different species because of the lack of useful phenotypic differential tests at that time [17,18,21]. A reference strain has been proposed for each genomovar and they have been deposited in culture collections and were publicly available. However, several species have been proposed in recent years that are phylogenetically closely related to *P. stutzeri*, but the gv reference strains have not been considered in the discussions. As a consequence, *S. chloritidismutans* (gv3), *S. kunmingensis* (gv3), *P. nitrititolerans* (gv8), and *S. zhaodongensis* (gv20) are species coincident to some previously reported genomovar of *P. stutzeri* (indicated in brackets). The main phenotypic difference between *S. chloritidismutans* and *P. stutzeri* was that the original strain AW-1T [24] was not able to denitrify but it was demonstrated later that denitrifying variants could be easily obtained as discussed later [25]. No set of clear physiological or biochemical tests can differentiate *S. chloritidismutans* from other *Stutzerimonas* species due to the phenotypic variability of the species in the genus. Nevertheless, the phylogenomic species 3, including *S. chloritidismutans* strains, can be easily differentiated phenotypically from strains in the other species or pgs by their main protein profiles obtained by MALDI TOF mass spectrometry [22] and by gene partial sequencing of housekeeping genes, like the *rpoD* gene [26]. Due to the superior value of molecular characteristics over the biochemical tests reached in recent years for the delineation of species, we propose that phylogenomic homogeneous groups defined in the present study as pgs should be considered representatives of true species yet to be described. To propose them as novel species, a complete phenotypic characterization is necessary, even though the biochemical tests do not always allow a clear phenotypic species differentiation.

## 5. Taxonomic Conclusions

As a general conclusion, we propose the assignation to the genus *Stutzerimonas* of all strains in the 49 phylogenomic species described in the present study. We describe two novel species within the genus *Stutzerimonas* and we emend the description of *S. perfectomarina* and *S. chloritidismutans* with data not included in the original descriptions. Furthermore, we propose that *S. kunmingensis* is a later heterotypic synonym of *S. chloritidismutans*. The genome of *P. tarimensis* has not been sequenced yet, but a multilocus sequence analysis demonstrated that this species belongs to the *P. stutzeri* phylogenetic group, close to *S. balearica* and *S. azotifigens* [27]. The species proposed to be transferred to the genus *Stutzerimonas* are summarized in Table 6.

### 5.1. Emended Description of S. perfectomarina (pgs2)

The name “*Pseudomonas perfectomarinus*” was proposed by ZoBell and Upham in 1944 in the description of sixty new marine bacterial species [33] but was not included on the Approved Lists of Bacterial Names. It was described as motile by means of one to several flagella at each pole, a denitrifier, and able to hydrolyze starch. Later on, Baumann et al. in 1983 [30] revived and corrected the name as “*Pseudomonas perfectomarina*”. The type strain and only member of the species was strain ATCC 14405T. It was found non-motile and this characteristic was emphasized by its assignation to a distinct species within the genus *Pseudomonas*. This strain has been considered a model organism for denitrification studies [34]. Experimental DNA-DNA homology studies demonstrated that the closest *Pseudomonas* to strain ZoBell were two *P. stutzeri* strains with 60–61% hybridization values that are on the borderline for species circumscription, but the strain was transferred to *P. stutzeri* by Döhler et al. [35]. The lack of motility by strain ZoBell was studied in our laboratory [36]. After three passages on semisolid tryptone agar, a motile revertant strain was obtained with over 80% of flagellated cells, and the DNA relatedness to the genomovar 2 reference strain of *P. stutzeri* (CCUG 44592=ATCC 17591) clarified the taxonomy of strain ZoBell. Genome sequence confirmed these results [37]. This is an example of how phenotypic properties have to be taken cautiously for classification. Phylogenomic species 2 contains four strains of quite different habitats and geographical origins. Another three well-studied strains by Stanier et al. [38] and Rosselló et al. [17] also belong to pgs2: ATCC 17587 (=Stanier 220=AB180=LMG 5838), ATCC 17592, and ATCC 17595. The strains have been isolated from marine samples, clinical specimens, *Bos Taurus* feces, and indoor dust. We propose the name *Stutzerimonas perfectomarina* for strains included in pgs2, with strain ZoBell as the type strain. The species description considering characteristics of seven strains obtained in this and previous studies is given in Table 3.

### 5.2. Emended Description of S. chloritidismutans (pgs3) and Synonymy with P. kunmingensis

*Pseudomonas chloritidismutans* was described by Wolterink et al. in 2002 [24]. The species description was based on the characterization of a single strain, although in the same publication it is stated that strain DSM 50227 of *P. stutzeri* (pgs3) and *P. chloritidismutans* AW-1 T are related at the species level. In the species description, only the characteristics of strain AW-1T are given, but not those of strain DSM 50227, nor for other strains of pgs3 known at that time. The only phenotypic trait that differentiated strain AW-1T from the rest of the strains in pgs·3 was the ability to grow anaerobically with chlorate and its inability to grow with nitrate. In simple adaptation experiments, Cladera et al. [25] demonstrated that variants of strain AW-1T can be obtained, in which the nitrate reductase activity is induced. This result was corroborated later by Mehboob et al. [39]. Chlorite-respiration is a property exclusive to strain AW-1T among strains of pgs3 and can be explained as the result of horizontal transfer events as suggested in other examples by Youngblut et al., [40]. In view of the results obtained in the present work, we propose to include all strains known in pgs3 in the same species as strain AW-1T and that strains previously assigned to *P. kunmingensis* have to be transferred to *S. chloritidismutans* because *P. kunmingensis* [32] is a later synonym. Strains of this species have been isolated from soil and marine waters and demonstrate a high potential to degrade contaminants. In previous studies, it was demonstrated that putrescin and spermidine were the most abundant polyamines in strains of pgs3 [41] and members of pgs3 can be differentiated from the rest of pgs by the main protein pattern obtained with MALDI-TOF mass spectrometry [22]. As consequence, the description of *S. chloritidismutans* species has to be emended and the species description is given in Table 2.

### 5.3. Description of S. frequens sp. nov. (pgs5)

Strains of pgs5 are widely distributed in the environment and have been isolated mainly from aquatic (marine, groundwater, and waste waters) from landfill or contaminated soil and associated with the rhizosphere (Table S1). None of them has been isolated from clinical specimens, but one was found in a clinical setting (an alcohol foam dispenser in a hospital intensive care unit). Strain DNSP21T (=CCUG 44521= DSM 6082= LMG 18520) is the reference strain of pgs5 and the type strain of the newly proposed species. Phenotypic and chemotaxonomic characteristics are given in Table 4 and Figure S5. *S. frequens* strains can be differentiated phenotypically from other species in the genus by the main protein profiles obtained with MALDI-TOF MS and by molecular methods based on the *rpoD* gene sequence analysis.

### 5.4. Description of S. degradans sp. nov. (pgs7)

Strains of pgs7 have been isolated mainly from soil and contaminated anaerobic habitats and are able to degrade aromatic contaminants, even under anoxic conditions with nitrate. None is associated with clinical specimens (Table S1). Strain DSM 50238T is the reference strain of pgs7 and was isolated from the soil as a denitrifier by Palleroni. DSM 50238T (=ATCC 17832=ICPB 2737-419=CCUG 44596=LMG 14935=CIP 107692) is the type strain of the newly proposed species. Phenotypic and chemotaxonomic characteristics are given in Table 5 and Figure S5. *S. degradans* strains can be differentiated phenotypically from other species in the genus by the main protein profiles obtained with MALDI-TOF MS and by molecular methods based on the *rpoD* gene sequence analysis.

## Figures and Tables

**Table 1:** Species type strains, genomovar reference strains, and related strains analyzed in the present study.

Species Names Proposed in the Current Taxonomy (1)	Strain	Assignation to *P. stutzeri* Genomovar	GTDB Taxonomy	Proposed Species Assignation to *Stutzerimonas*
*S. azotifigens*	6H33bT; ATCC BAA-1049T; DSM 17556T; JCM 12708T	-	Pseudomonas_N azotifigens	*S. azotifigens*
*S. balearica*	CCUG 44487T; CIP 105297T; DSM 6083; SP1402T	gv6 ref	Pseudomonas_A balearica	*S. balearica*
*S. chloritidismutans*	ATCC BAA-443T; AW-1T; DSM 13592T; NBRC 102227T	gv3	Pseudomonas_A chloritidismutans	*S. chloritidismutans*
*S. kirkiae*	FRB 229T; LMG 31089T; NCPPB 4674T; P4CT	-	Pseudomonas_Q kirkiae	*S. kirkiae*
*S. kunmingensis*	CGMCC 1.12273T; DSM 25974T; HL22-2 T	gv3	Pseudomonas_A kunmingensis	*S. chloritidismutans*
*P. nitrititolerans*	CGMCC 1.13874T; GL14T; NBRC 113853T	gv8	Pseudomonas_A nitrititolerans	*S. nitrititolerans*
*S. nosocomialis*	A31/70T; CCUG 73638T; CECT 9881T	-	Pseudomonas_R nosocomialis	*S. nosocomialis*
*P. perfectomarina*	ZoBellT; ATCC 14405T; LMG 14935T	gv2	Pseudomonas_A stutzeri_AC	*S. perfectomarina*
*S. stutzeri*	ATCC 17588T; CCUG 11256T; CFBP 2443T; CIP 103022T; DSM 5190T; IFO 14165T; JCM 5965T; LMG 11199T; NBRC 14165T	gv1 ref	Pseudomonas_A stutzeri	*S. stutzeri*
*P. tarimensis*	CCTCC AB 2013065T; KCTC 42447T; MA-69T	-	not found	*S. tarimensis*
*S. urumqiensis*	ACCC 60124T; JCM 32830T; T3T	-	Pseudomonas_N sp003640395	*S. urumqiensis*
*S. xanthomarina*	CCUG 46543T; DSM 18231T; JCM 12468T; KMM 1447T; NRIC 617T	-	Pseudomonas_A xanthomarina	*S. xanthomarina*
*S. zhaodongensis*	ACCC 06362T; DSM 27559T; NEAU-ST5-21T	gv20	Pseudomonas_A zhaondongensis	*S. zhaodongensis*
“*P. lopnurensis*”	AL-54T; CCTCC AB 2013066T; JCM 19136T; NRRL B-59987T	-	not found	*“S. lopnurensis”*
“*P. saudiphocaensis*”	20_BNT	-	Pseudomonas_A saudiphocaensis	*“S. saudiphocaensis”*
“*P. songnenensis*”	ACCC 06361T; DSM 27560T; NEAU-ST5-5T	-	Pseudomonas_A songnenensis	*“S. songnenensis”*
*P. stutzeri*	CCUG 44592; ATCC 17591	gv2 ref	Pseudomonas_A stutzeri_AC	*S. perfectomarina*
*P. stutzeri*	DSM 50227; CCUG 711, NRRL B-927, NCTC 10475, LMG 1228, CIP 59.54, DSM 50227, ATCC 11607, ICPB 2445, NCIB 9721	gv3 ref	Pseudomonas_A chloritidismutans	*S. chloritidismutans*
*P. stutzeri*	19SMN4; CCUG 44593; DSM 6084; LMG 18521	gv4 ref	Pseudomonas_A stutzeri_A	*Stutzerimonas* pgs4
*P. stutzeri*	DNSP21; CCUG 44521; DSM 6082; LMG 18520	gv5 ref	Pseudomonas_A sp003205815	*S. frequens*
*P. stutzeri*	DSM 50238	gv7 ref	Pseudomonas_A sp000765155	*S. degradans*
*P. stutzeri*	JM300	gv8 ref	Pseudomonas_A stutzeri_AG	*S. nitrititolerans*
*P. stutzeri*	KC; DSM 7136	gv9 ref	Pseudomonas_A stutzeri_AA	*Stutzerimonas* pgs9
*P. stutzeri*	CLN100	gv10 ref	not found	*Stutzerimonas* pgs10
*P. stutzeri*	28a50	gv11 ref	Pseudomonas_A stutzeri_D	*Stutzerimonas* pgs11
*P. stutzeri*	28a39	gv12 ref	Pseudomonas_A stutzeri_AI	*Stutzerimonas* pgs12
*P. stutzeri*	28a22	gv13 ref	not found	*Stutzerimonas* pgs13
*P. stutzeri*	28a3	gv14 ref	Pseudomonas_A stutzeri_AI	*Stutzerimonas* pgs14
*P. stutzeri*	4C29	gv15 ref	Pseudomonas_A stutzeri_AB	*Stutzerimonas* pgs15
*P. stutzeri*	24a13	gv16 ref	Pseudomonas_A stutzeri_AH	*Stutzerimonas* pgs16
*P. stutzeri*	24a75	gv17 ref	Pseudomonas_A stutzeri_AF	*Stutzerimonas* pgs17
*P. stutzeri*	MT-1; CCUG 50545	gv18 ref	Pseudomonas_A sp004010935	*Stutzerimonas* pgs18
*P. stutzeri*	CCUG 46542	gv19 ref	not found	*Stutzerimonas* pgs19
*P. stutzeri*	PE	gv20 ref	not found	*S. zhaodongensis*
*P. stutzeri*	A563/77	gv21 ref	not found	*Stutzerimonas* pgs21
*P. stutzeri*	V81	gv22 ref	not found	*Stutzerimonas* pgs22
*P. stutzeri*	KOS6		Pseudomonas_A stutzeri_B	*Stutzerimonas* pgs23
*P. stutzeri*	RCH2		Pseudomonas_A stutzeri_AE	*Stutzerimonas* pgs24
*P. stutzeri*	RM2		not found	*Stutzerimonas* pgs25
*P. stutzeri*	TS44		Pseudomonas_A stutzeri_C	*Stutzerimonas* pgs26
*P. azotifigens*	115A1		Pseudomonas_R azotifigens	*Stutzerimonas* pgs27
*Pseudomonas sp.*	ABC1		Pseudomonas_Q sp013395055	*Stutzerimonas* pgs28
*P. stutzeri*	95A6		Pseudomonas_A stutzeri_AK	*Stutzerimonas* pgs29
*P. stutzeri*	NT0128		Pseudomonas_A stutzeri_E	*Stutzerimonas* pgs30
	Q2-TVG-42		not found	*Stutzerimonas* pgs31
*Pseudomonas sp.*	SST3		Pseudomonas_A sp003325755	*Stutzerimonas* pgs32
*P. stutzeri*	PM101005		Pseudomonas_A stutzeri_R	*Stutzerimonas* pgs33
*P. stutzeri*	A4		not found	*Stutzerimonas* pgs34
*P. stutzeri*	SD25545	gv12	not found	*Stutzerimonas* pgs35 (former gv12)
*P. stutzeri*	99A1		Pseudomonas_A sp004331835	*Stutzerimonas* pgs36
*P. stutzeri*	SDM-LAC		Pseudomonas_A stutzeri_H	*Stutzerimonas* pgs37
*P. stutzeri*	AN10	gv3	Pseudomonas_A stutzeri_U	*S. chloritidismutans*
*P. stutzeri*	28a24	gv11	Pseudomonas_A stutzeri_D	*Stutzerimonas* pgs11
*P. stutzeri*	NF13	gv19	Pseudomonas_A stutzeri_G	*Stutzerimonas* pgs19

(1) Quotation marks: not validated names. ref: *P. stutzeri* genomovar reference strain.

**Table 2:** Emended description of *Stutzerimonas chloritidismutans*.

Species Name	*Stutzerimonas chloritidismutans*
Species status	Validly published under the ICNP
Species etymology	chlo.ri.ti.dis.mu.tans. Gr. fem. n. chlôris, a female yellow bird; N.L. pres. part. dismutans, splitting; N.L. part. adj. *chloritidismutans*, chlorite-splitting
Designation of the type strain	AW-1^T^
Strain collection numbers	ATCC BAA-443; DSM 13592; NBRC 102227
16S rRNA gene accession number	AY017341 (1501 nt)
Alternative housekeeping genes	*gyrB* gene [AJ880092.2] and *rpoD* gene [AJ880091.1]
Genome accession number	AOFQ00000000.1
Genome status	Draft
Genome size	5.0 Mb
GC mol%	62.5
Country of origin	Netherlands
Region of origin	Wageningen
Other strains	CCUG 29243; ST-9; DSM 25974T
Date of isolation	2002
Source of isolation	Biomass of an anaerobic bioreactor treating chlorate- and bromate-polluted wastewater
Sampling date	2002
Heterotypic synonyms	*Pseudomonas stutzeri* gv3 (DSM 50227), *Pseudomonas kunmingensis* DSM 25974T
Source of isolation of non-type strains	Clinical specimens, before 1952 (DSM 50227); phosphate rock powder from a phosphate mining field; China; Yunnan Province, suburb of Kunming; isolated: 20.10.2009 (DSM 25974T); soil; sea water; contaminated marine sediment
Growth medium, incubation conditions used for standard cultivation	Luria-Bertani medium (LB) at 30 °C
Gram stain	Negative
Cell shape	Rods
Cell size (length or diameter)	Cells are 0.5–2 µm in size
Motility	Motile by a single polar flagellum
Colony morphology	Colonies growing in anaerobic agar are circular and pale orange. Colonies on nutrient broth plates are wrinkled, coherent, and have a pale brown or sometimes reddish, brown color. Smooth colonies may be produced.
Growth temperature range	10–37 °C
Temperature optimum	30 °C
Growth pH range	7–9
Growth NaCl range	0–4
Metabolism	Facultatively aerobic, strictly respiratory, able to grow anaerobically with nitrate or chlorate (some strains)
BIOLOG GEN III positive tests for the type strain	Substrates oxidized: alfa-D-glucose, Tween 40, dextrin, D-mannitol, methyl-pyruvate, gamma-amino-butyric acid, D-maltose, D-fructose, D-arabitol, L-alanine, alfa-hydroxy butyric acid, D-trehalose, D-gluconic acid, L-lactic acid, beta-Hydroxy-D-L-butyric acid, glycerol, L-aspartic acid, citric acid, alfa-keto-butyric acid, L-glutamic acid, alfa-keto glutaric acid, mucic acid, D-malic acid, propionic acid, L-pyroglutamic acid, L-malic acid, acetic acid, D-saccharic acid, formic acid, Growth: 1% NaCl, 1% sodium lactate, troleandomycin, lincomycin, vancomycin nalidixic acid, aztreonam, 4% NaCl, fusidic acid, rifamycin SV, guanidine HCl, tetrazolium violet, lithium chloride, sodium butyrate, D-serine, minocycline, niaproof 4, tetrazolium blue, potassium tellurite.
BIOLOG GEN III negative tests for the type strain	Not able to oxidize: D-raffinose, D-sorbitol, gelatine, pectin, p-hydroxy, phenylacetic acid, alfa D-lactose, D-mannose, glycyl-L-proline, D-galacturonic acid, D-melibiose, D, lactic acid methyl ester, beta-methyl-D-glucoside, D-galactose, myo-inositol, L-arginine, D-cellobiose, D-salicin, 3-methyl glucose, D-glucuronic acid, D-gentiobiose, N-acetyl-D-glucosamine, D-glucose-6-PO4, glucuronamide, acetoacetic acid, sucrose, N-acetyl, beta-D-mannosamine, L-fucose, D-fructose-6-PO4, L-histidine, turanose, N-acetyl-D-galactosamine, L-rhamnose, D-aspartic acid, quinic acid, stachyose, N-acetyl-neuraminic acid, inosine, D-serine, L-Serine, pH 5
BIOLOG GEN III differential tests for strains DSM 50227 and DSM 25974T	Oxidized substrates: D-galactonic acid lactone, D-glucuronic acid, mucic acid, L-pyroglutamic acid, D-saccharic acid.
API 20NE positive tests for the type strain	Reduction of nitrate to nitrite and nitrite to N2, glucose, mannitol, maltose, gluconate, caprate, malate, citrate.
API 20NE negative tests for the type strain	Indole production, glucose fermentation. Presence of arginine dihydrolase, urease, hydrolysis of aesculin, hydrolysis of gelatin, beta-galactosidase- Assimilation of: Arabinose, Mannose, N-acetyl-D-glucosamine, Adipate, Phenylacetate.
API 20NE differential tests for strains DSM 50227 and DSM 25974T	Assimilation of Mannitol
Energy metabolism of strains in the species	Chemoorganotroph, strictly oxidative, anaerobic with nitrate or chlorate. Thiosulfate oxidation in 2 strains.
Oxidase	Positive
Catalase	Positive
Pigment production on King A and King B media	Negative
Major fatty acids (mean values of 10 strains) [17]	0.10% decanoate (10:0); 2.79% 3-hydroxydecanoate (3OH 10:0); 0.00% 10-methyldodecanoate (a-13:0); 7.69% dodecanoate (12:0); 2.64% 3-hydroxydodecanoate (3OH 12:0); 0.98% tetradecanoate (14:0); 0.00% pentadecanoate (15:0); 28.77% cis 9 hexadecanoate (16:1); 21.45% hexadecanoate (16:0); 0.61% 15 methylhexadecenoate (17:0); 1.26% cis 9,10 methylenehexadecanoate (17:0c); 29.58% cis 9,11 octadecenoate (18:1); 1.46% octadecanoate (18:0); 1.45% cis 9,10 methyleneoctadecanoate (19:0c).
Biosafety level	1
Habitat	Soil, wastewater sludge, marine sediment, rhizosphere, clinical specimens
Biotic relationship	Free-living
Known pathogenicity	None

API 20NE data for strain AW-1T were obtained in our laboratory. Results for other strains were retrieved from BacDive.

**Table 3:** Emended description of *Stutzerimonas perfectomarina* comb. nov.

Species Name	*Stutzerimonas perfectomarina*
Species status	nom. rev.
Species etymology	per.fec.to.ma.rí.na. L. masc. perf. part. *perfectus*, complete, perfect; L. fem. adj. *marina*, of the sea, marine; N.L. fem. adj. *perfectomarina*, completely marine
Previous validly published names (Synonyms)	*Bacterium stutzeri* (Lehmann and Neumann 1896), *Pseudomonas perfectomarinus* (ZoBell and Upham 1944), *Pseudomonas perfectomarina* (Baumann et al., 1983) trasferred to *Pseudomonas stutzeri* (Döhler et al., 1983), member of genomovar 2 of *P. stutzeri* (Rosselló-Móra et al., 1993)
Designation of the type strain	ZoBell 632
Type strain collection numbers	Baumann 218; ATCC 14405; CCUG 16156; CECT 4899; JCM 20778; LMG 2243
16S rRNA gene accession number	U26420 (1456 nt), U65012 (rRNA operon)
Alternative housekeeping genes	*gyrB* gene [AJ617564], and *rpoD* gene [AGSL01000000]
Genome accession number	AGSL01000000
Genome status	Draft
Genome size (pb)	4.7 Mb
GC mol%	61.4
Country of origin	USA
Region of origin	California, Pacific Ocean
Date of isolation	1944
Source of isolation	Marine water
Sampling date	1944
Other strains	Stanier 224 (=ATCC 17591, =CIP 107689, =CCUG 44592), Stanier 220a (=ATCC 17587), Stanier 225a (=ATCC 17592), Stanier 228a (=ATCC 17595)
Source of isolation of non-type strains	Clinical specimens, indoor dust and *Bos taurus* feces
Growth medium, incubation conditions used for standard cultivation	Luria-Bertani medium (LB) at 30 °C
Gram stain	Negative
Cell shape	Straight or slightly curved rod
Cell size (length or diameter)	1.1 to 1.9 µm long and 0.5 to 0.7 µm wide
Motility	Motile by a single polar flagellum
Colony morphology	Colonies are mucous, beige-colored, from round to oval, with irregular margins. 2–3 mm in diameter after growth for 48 h, 2–5 mm after 72 h at 30 °C on LB plates. Smooth and rough colonial morphologies may be produced.
Growth temperature range	4–40 °C
Temperature optimum	30 °C
Growth pH range	7–10
Growth NaCl range	0–8%
Metabolism	Facultatively aerobic, strictly respiratory, able to grow anaerobically with nitrate
BIOLOG GEN III positive tests for the type strain	Growth: 1–4–8% NaCl, 1% sodium lactate, troleandomycin, lincomycin, vancomycin, nalidixic acid, aztreonam, pH 6, fusidic acid, rifamycin SV, guanidine HCl, tetrazolium violet, lithium chloride, sodium butyrate, D-serine, minocycline, niaproof 4, tetrazolium blue, potassium tellurite. Substrates oxidized: alfa-D-glucose, tween 40, dextrin, D-galacturonic acid, methyl pyruvate, D-maltose, D-fructose, D-arabitol, L-alanine, D-galactonic acid lactone, alfa-hydroxy butyric acid, L-lactic acid, beta-hydroxy-D, L-butyric acid, glycerol, L-aspartic acid, D-glucuronic acid, citric acid, alfa-keto-butyric acid, L-glutamic acid, alfa-keto glutaric acid, mucic acid, D-malic acid, propionic acid, L-pyroglutamic acid, L-malic acid, acetic acid, D-saccharic acid, and formic acid.
BIOLOG GEN III negative tests for the type strain	No growth at pH 5. Not able to oxidize D-raffinose, D-sorbitol, gelatin, pectin, p-hydroxy-phenylacetic acid, alfa-D-lactose, D-mannose, D-mannitol, glycyl-L-proline, gamma-amino-butyric acid, D-melibiose, D-lactic acid methyl ester, D-trehalose, beta-methyl-D-glucoside, D-galactose, myo-Inositol, L-arginine, D-gluconic acid, D-cellobiose, D-salicin, 3-methyl glucose, gentiobiose, N-acetyl-D-galactosamine, D-fucose, D-glucose-6-PO4, acetoacetic acid, sucrose, N-acetyl-beta-D-mannosamine, L-fucose, D-fructose-6-PO4, D-fructose-6-PO4, turanose, N-acetyl-D-galactosamine, L-rhamnose, D-aspartic acid, quinic acid, stachyose, N-acetyl-neuraminic acid, inosine, N-serine, and bromo-succinic acid.
BIOLOG GEN III differential tests for strain ATCC 17591	Oxidation of D-mannitol, D-galactonic acid, D-trehalose, D-gluconic acid, mucic acid, D-saccaric acid, and formic acid.
API 20NE positive tests for the type strain	Reduction of nitrate to nitrite and nitrite to N_2_, the assimilation of glucose, caprate, malate, and citrate.
API 20NE negative tests for the type strain	Indole production, glucose fermentation, arginine dihydrolase, urease, aesculin hydrolysis, hydrolysis of gelatin, and beta-galactosidase. Assimilation of arabinose, mannose, mannitol, N-acetyl-D-glucosamine, maltose, gluconate, adipate, phenyl-acetate.
API 20NE differential tests for strain ATCC 17591	Mannitol, maltose, gluconate.
Energy metabolism	Chemoorganotrophic, denitrifier with nitrate, strictly oxidative
Oxidase	Positive
Catalase	Positive
Pigment production on King A and King B media	Negative
Major fatty acids (mean values of 5 strains) [17]	0.06% decanoate (10:0); 2.92% 3-hydroxydecanoate (3OH 10:0); 0.00 10-methyldodecanoate (a-13:0); 9.33% dodecanoate (12:0); 2.77% 3-hydroxydodecanoate (3OH 12:0); 0.72% tetradecanoate (14:0); 0.03% pentadecanoate (15:0); 25.55% cis 9 hexadecanoate (16:1); 20.93% hexadecanoate (16:0); 0.4% 15 methylhexadecenoate (17:0); 0.26% cis 9,10 methylenehexadecanoate (17:0c); 34.64% cis 9,11 octadecenoate (18:1); 1.80% octadecanoate (18:0); 0.46% cis 9,10 methyleneoctadecanoate (19:0c).
Biosafety level	1
Habitat	Marine, human urine
Biotic relationship	Free-living
Known pathogenicity	Not known pathogenicity but present in clinical specimens

API 20NE data for strain ATCC 14405T were obtained in our laboratory. Results for other strains were retrieved from BacDive.

**Table 4:** Description of *Stutzerimonas frequens* sp. nov.

Species Name	*Stutzerimonas frequens*
Species status	sp. nov.
Species etymology	fre’quens. L. adj. *frequens*, frequently isolated from different habitats
Designation of the type strain	DNSP21
Type strain collection numbers	DSM 6082; LMG 1852; CCUG 44594
16S rRNA gene accession number	U26414.1 (1456 nt)
Alternative housekeeping genes	*gyrB* gene [AJ620493.1] and *rpoD* gene [AJ631335.1]
Genome accession number	POUJ01000000 and NZ CP065720
Genome status	Draft
Genome size (pb)	4.4 Mb
GC mol%	58.7
Country of origin	Spain
Region of origin	Mallorca (Santa Ponça, wastewater treatment plant)
Date of isolation	1988
Source of isolation	Wastewater
Sampling date	1988
Geographic location	Wastewater treatment plant, Santa Ponça,
Latitude	39°31′18.8″ N
Longitude	2°30′45.4″ E
Other strains	St103, St104, HI00D01, R2A2, KOL14.W.45.16, KOL14.W.90.4, KOL14.W.5.3, KOL14.W.20.12, KOL14.W.90.9, THAF7b, TPA3, ChG 5-2
Source of isolation of non-type strains	Pacific Ocean, near Hawaii (HI00D01); groundwater South-west Taiwan; hydrothermal vent Greece (KOL14.W.45.16, KOL14.W.90.4, KOL14.W.5.3, KOL14.W.20.12, KOL14.W.90.9); microplastics (THAF7b); soil (TPA3); *Phragmites australis* (St103), and *Spartina patens* (St104) rhizosphere; Black Sea 120m depth (ChG 5-2).
Growth medium, incubation conditions used for standard cultivation	Luria-Bertani medium (LB) at 30 °C
Gram stain	Negative
Cell shape	Rod
Cell size (length or diameter)	2 − 5 × 0.8 − 1 µm
Motility	Motile, one polar inserted flagellum
Colony morphology	Colonies are round, dry, and wrinkled, beige-colored, with irregular margins. Its size was 2–3 mm to 3–5 mm in diameter after growth for 48 h at 30 °C on LB plates. Colonies strongly attached to the agar.
Growth temperature range	4–37 °C
Temperature optimum	30 °C
Growth pH range	6–10
Growth NaCl concentration	0–8
Metabolism	Facultatively aerobic, strictly respiratory, able to grow anaerobically with nitrate
BIOLOG GENIII positive tests for the type strain	The strains have the ability to oxidize: alfa-D-glucose, Tween 40, dextrin, D-mannitol, methyl-pyruvate, gamma-amino-butyric acid, D-maltose, D-fructose, L-alanine, alfa-hydroxy-butyric acid, D-gluconic acid, L-lactic acid, beta-hydroxy-D, L-butyric acid, glycerol, citric acid, alfa-keto-butyric acid, L-glutamic acid, alfa-keto glutaric acid, D-malic acid, propionic acid, L-malic and acid, acetic acid, bromo-succinic acid, formic acid. Able to grow at 1% NaCl, 4% NaCl 8% NaCl, 1% sodium lactate, troleandomycin, lincomycin, vancomycin, nalidixic acid, aztreonam, pH 6, fusidic acid, rifamycin SV, guanidine HCl, tetrazolium violet, lithium chloride, sodium butyrate, D-serine, minocycline, niaproof 4, potassium tellurite, sodium bromate.
BIOLOG GENIII negative tests for the type strain	The strains were unable to oxidize: D-raffinose, D-sorbitol, gelatin, pectin, p-hydroxy-phenylacetic acid, alfa-D-lactose, D-mannose, glycyl-L-proline, D-galacturonic acid, D-melibiose, D-arabitol, D-galactonic acid lactone, D-lactic acid methyl ester, D-trehalose, beta-methyl-D-glucoside, D-galactose, myo-inositol, L-arginine, D-cellobiose, D-salicin, 3-methyl glucose, L-aspartic acid, D-glucuronic acid, gentiobiose, N-acetyl-D-glucosamine, D-fucose, D-glucose-6-PO4, acetoacetic acid, sucrose, N-acetyl-beta-D-mannosamine, L-fucose, D-fructose-6-PO4, L-histidine, mucic acid, turanose, N-acetyl-D-galactosamine, L-rhamnose, D-aspartic acid, L-pyroglutamic acid, quinic acid, stachyose, N-acetyl-neuraminic acid, inosine, D-serine, L-serine, D-saccharic acid Unable to grow at pH 5.
API 20NE positive tests for the type strain	Reduction of nitrate to nitrite and nitrite to N_2,_ assimilation of glucose, maltose, gluconate, caprate, malate, and citrate.
API 20NE negative tests for the type strain	Indole production, glucose fermentation, arginine dihydrolase, urease, hydrolysis of aesculin, hydrolysis of gelatine, beta-galactosidase. No assimilation of arabinose, mannose, mannitol, N-acetyl-D-glucosamine, adipate, and phenylacetate.
Energy metabolism	Chemoorganotroph, denitrifier, strictly oxidative
Oxidase	Positive
Catalase	Positive
Pigment production on King A and King B media	Negative
Major fatty acids of the type strain [17]	0.00% decanoate (10:0); 3.42% 3-hydroxydecanoate (3OH 10:0); 0.00% 10-methyldodecanoate (a-13:0); 8.46% dodecanoate (12:0); 2.88% 3-hydroxydodecanoate (3OH 12:0); 0.05% tetradecanoate (14:0); 0.00% pentadecanoate (15:0); 24.78% cis 9 hexadecanoate (16:1); 21.57% hexadecanoate (16:0); 0.40% 15 methylhexadecenoate (17:0); 0.28% cis 9,10 methylenehexadecanoate (17:0c); 35.79% cis 9,11 octadecenoate (18:1); 1.04% octadecanoate (18:0); 0.32% cis 9,10 methyleneoctadecanoate (19:0c).
Biosafety level	1
Habitat	Marine water, ground water, hydrothermal vent, and soil
Biotic relationship	Free-living
Known pathogenicity	None

API 20NE data were retrieved from BacDive.

**Table 5:** Description of *Stutzerimonas degradans* sp. nov.

Species Name	*Stutzerimonas degradans*
Species status	sp. nov.
Species etymology	de.gra’dans. L. part adj. *degradans*, returning to the original order, referring to the ability of the type strain to degrade contaminants
Designation of the type strain	DSM 50238
Type strain collection numbers	ATCC 17832, ICPB 2737-419, CCUG 44596, LMG 14935, CIP 107692, Stanier 419
16S rRNA gene accession number	U26416.1 (1456 bp)
Alternative housekeeping genes	*gyrB* gene [AJ631262.1] and *rpoD* gene [AJ631339.1]
Genome accession number	CP065721
Genome status	Complete
Genome size (pb)	4.0 Mb
GC mol%	64.5
Country of origin	USA
Region of origin	Berkeley, California
Other strains	AER2.7, 4FB3, 2FB7A, Chol1, DCP-Ps1, KF716
Other strains in this pgs	HMP271, KF716, DCP-Ps1, PheN2, JL972, XL272, MM2020_2, D-134-1
Date of isolation	Before 1966
Source of isolation	Isolated from the soil by L-(+)-tartrate enrichment
Geographic location	California
Region of origin	Berkeley
Source of isolation of non-type strains	Aircraft-oil contaminated soil (AER2.7); estuarine sediment (4FB3, 2FB7A); soil (Chol1, DCP-Ps1, KF716); coal cutting; lab enrichment culture in anaerobic medium (HMP271), biphenyl contaminated soil (KF716), dichlorophenol enrichment culture inoculated with agricultural soil (DCP-Ps1), anaerobic environments (PheN2), aquatic sediment (JL972), cucumber rhizosphere (XL272), freshwater sediment (MM2020_2), soil (D-134-1)
Growth medium, incubation conditions used for standard cultivation	Luria-Bertani medium (LB) at 30 °C
Gram stain	Negative
Cell shape	Rods
Cell size (length or diameter)	3.5 − 4.8 × 0.3 − 0.7 µm
Motility	Motile, one polar inserted flagellum
Colony morphology	Freshly isolated strains: rough, wrinkled, dry and coherent, light brown in color. Colonies are round, beige color. Its size is 2–3 mm in diameter after growth for 48 h at 30 °C on LB plates. Colonies with regular margins and translucent may be produced.
Growth temperature range	10–42 °C
Temperature optimum	30 °C
Growth pH range	6–10
Growth NaCl range	0–8%
Metabolism	Facultatively aerobic, strictly respiratory, able to grow anaerobically with nitrate
BIOLOG GENIII positive tests for the type strain	Oxidized substrates: alfa-D-glucose, Tween 40, dextrin, methyl pyruvate, gamma-amino-butyric acid, D-maltose, L-alanine, alfa-hydroxy butyric acid, L-lactic acid, beta-hydroxy-D, L-butyric acid, glycerol, L-aspartic acid, citric acid, alfa-keto butyric acid. L-glutamic acid, alfa-keto glutaric acid, D-malic acid, propionic acid, L-malic acid, acetic acid, L-serin, bromo-succinic acid. Growth with 1–4–8% NaCl, pH 6, 1% sodium lactate, troleandomycin, lincomycin, vancomycin, nalidixic acid, aztreonam, fusidic acid, rifamycin SV, guanidine HCl, tetrazolium violet, lithium chloride, sodium butyrate, D-serine, minocycline, niaproof 4, tetrazolium blue and potassium tellurite.
BIOLOG GENIII negative tests for the type strain	Not oxidized substrates: D-raffinose, D-sorbitol, gelatin, pectin, p-hydroxy-phenylacetic acid, alfa-D-lactose, D-mannose, D-mannitol, glycyl-L-proline, D-galacturonic acid, D-melibiose, D-fructose, D-arabitol, D-galactonic acid lactone, D-lactic acid methyl ester, D-trehalose, beta-methyl-D-glucoside, D-galactose, myo-inositol, L-arginine, D-gluconic acid, D-cellobiose, D-salicin, 3-methylglucose, D-glucuronic acid, gentiobiose, N-acetyl-D-glucosamine, D-glucose-6-PO4, glucuronamide, acetoacetic acid, N-acetyl-beta-D-mannosamine, L-fucose, D-fructose-6-PO4, L-histidine, mucic acid, turanose, N-acetyl-D-galactosamine, L-rhamnose, D-aspartic acid, L-pyroglutamic acid, quinic acid, stachyose, N-acetyl-neuraminic acid, inosine, D-serine, and D-saccharic acid, formic acid. Strains were unable to grow at pH 5, nor with sodium bromate.
API 20NE positive tests for the type strain	Reduction of nitrate to nitrite and nitrite to N_2,_ the assimilation of glucose, maltose, caprate, malate, and citrate.
API 20NE negative tests for the type strain	Indole production, glucose fermentation, arginine dihydrolase, urease, hydrolysis of aesculin, Hydrolysis of gelatin, beta-galactosidase, arabinose, mannose, mannitol, and N-acetyl-D-glucosamine, gluconate, adipate, phenylacetate
Energy metabolism	Chemoorganotroph, denitrifier, strictly oxidative
Oxidase	Positive
Catalase	Positive
Pigment production on King A and King B media	Negative
Major fatty acids [17]	0.00% decanoate (10:0); 2.76% 3-hydroxydecanoate (3OH 10:0); 0.00 10-methyldodecanoate (a-13:0); 10.80% dodecanoate (12:0); 2.51% 3-hydroxydodecanoate (3OH 12:0); 1.04% tetradecanoate (14:0); 0.00% pentadecanoate (15:0); 29.60% cis 9 hexadecanoate (16:1); 22.47% hexadecanoate (16:0); 0.76% 15 methylhexadecenoate (17:0); 1.64% cis 9,10 methylenehexadecanoate (17:0c); 26.15% cis 9,11 octadecenoate (18:1); 1.37% octadecanoate (18:0); 0.89% cis 9,10 methyleneoctadecanoate (19:0c).
Biosafety level	1
Habitat	Soil, estuarine sediment
Biotic relationship	Free-living
Known pathogenicity	None

API 20NE data were retrieved from BacDive.

**Table 6:** Description of the new combinations in the genus *Stutzerimonas* gen. nov.

New Name Combination and Etymology	Basonym	Description	Type Strain and Culture Collection Numbers
*Stutzerimonas nitrititolerans* *nitritum,* nitrite; L. pres. part. *tolerans,* tolerating; N.L. part. adj. *nitrititolerans,* tolerating nitrite	*Pseudomonas nitrititolerans*	The description of this taxon is as given by Peng et al., 2019 [28]	CGMCC 1.13874T; GL14T; NBRC 113853T
*Stutzerimonas nosocomialis* no.so.co.mi.a’lis. N.L. neut. n. *nosocomium*, a hospital, infirmary; L. masc./fem. adj. suff. *-alis*, suffix used with the sense of pertaining to; N.L. masc./fem. adj. *nosocomialis*, pertaining to a hospital	*Pseudomonas nosocomialis*	The description of this taxon is as given by Mulet et al., 2019; and 2022 [23]	A31/70T; CCUG 73638T; CECT 9881T
*Stutzerimonas tarimensis* ta.rim.en’sis. N.L. masc./fem. adj. *tarimensis*, pertaining to Tarim basin in Xinjiang Uyghur autonomous region of China, where the type strain was isolated	*Pseudomonas tarimensis*	The description of this taxon is as given by Anwar et al., 2017 [27]	CCTCC AB 2013065T; KCTC 42447T; MA-69T
*Stutzerimonas urumqiensis* u.rum.qi.en’sis. N.L. masc./fem. adj. *urumqiensis*, pertaining to Urumqi, in Xinjiang Uyghur Autonomous Region of China, 45°5′6″ N, 94°58′36″ E, where the sample was collected	*Pseudomonas urumqiensis*	The description of this taxon is as given by Zou et al., 2019 [29]	ACCC 60124T; JCM 32830T; T3T
*Stuzerimonas perfectomarina* per.fec.to.ma.ri.na. L. masc. perf. Part. *perfectus*, complete, perfect; L. fem. adj. *marina*, of the sea, marine; N.L. fem. adj. *perfectomarina*, completely marine	*Pseudomonas perfectomarina*	The description of this taxon is as given by Bauman et al., 1983 [30] and by the present study	ZoBell 632T; Baumann 218T; ATCC 14405T; CCUG 16156T; CECT 4899T; JCM 20778T; LMG 2243T
*Stutzerimonas zhaodongensis* zhao.dong.en’sis. N.L. masc./fem. adj. *zhaodongensis*, pertaining to Zhaodong City, China, where the type strain was isolated	*Pseudomonas zhaodongensis*	The description of this taxon is as given by Zhang et al., 2015 [31]	ACCC 06362T; DSM 27559T; NEAU-ST5-21T
Heterotypic synonym:			
*Stutzerimonas chloritidismutans*	*Pseudomonas kunmingensis* (heterotypic synonym)	The description of this taxon is as given by Xie et al., 2014 [32] and the present study	ATCC BAA-443T; DSM 13592T; NBRC 102227T; AW-1T

## Data Availability

The Whole Genome Shot-gun projects for all strains sequenced have been deposited in DDBJ/ENA/GenBank under the accession numbers JAMOHM00000000—JAMOHZ00000000, JAMOIA00000000—JAMOIJ00000000, JAMO-JY00000000—JAMOJZ00000000, JAMQRT000000000 and JAMZSL000000000-JAMZSP000000000. The version described in this paper are the first version, numbers JAMOHM01000000—JAMOHZ01000000, JAMOIA01000000—JAMOIJ01000000, JAMOJY01000000—JAMOJZ01000000, JAMQRT010000000 and JAMZSL010000000-JAMZSP010000000.

## Supplementary Materials

The following supporting information can be downloaded at: https://www.mdpi.com/article/10.3390/microorganisms10071363/s1, Figure S1. Phylogenetic analysis was obtained at the GTDB-Anno tree website of representative strains in the *Pseudomonadaceae* (A) and in *Stutzerimonas* (B). Figure S2. Core-genome phylogeny based on the concatenated sequences of 666 core genes of 200 genomes including members of *Azotobacter*, *Azomonas,* and *Pseudomonas* species as outgroup. Tree was constructed using the maximum likelihood method. Bootstrap values of 100 replicates are indicated in the nodes with different symbols: circles indicate bootstrap values of 100, squares indicate values higher than 80%, and starts with values higher than 70%. Figure S3. Correspondence of the core-genome phylogeny and the GTDB taxonomy among 62 genomes of pgs representative strains and GTDB representative genomes. Sequences of 758 genes were concatenated and analyzed. Figure S4. UPGMA dendrogram based on the Jaccard index calculated from the phyletic pattern. * indicates the exceptions in the grouping pattern. Figure S5. Transmission electron micrograph of negatively stained cells of *S. frequens* strain DNSP21T (A) and *S. degradans* strain DSM 50238T (B) showing the polar inserted flagellum. Table S1. Strains in the genus *Stutzerimonas* and related species whose genomes have been sequenced, accession numbers, classification, and origin. Genome sequences obtained in this study are indicated. Reference and type strains are highlighted in bold. When several genomic sequences of the same strain in the databases were available, or when the sequences were obtained from equivalent strains from different culture collections, only the one marked with an asterisk was analyzed. Table S2. Morphological, physiological, and biochemical characteristics of the novel and emended species of *Stutzerimonas* proposed in the present study. Table S3. ANIb matrix calculated with the Jspecies program. Each cluster corresponds to a phylogenomic species; the species are ordered following the ANIb values. Groupings are coincident with the phylogenetic groupings obtained in the core-genome phylogeny. Genome sequences obtained in this study are indicated in bold. Supplementary information. Concatenated core-genome gene alignments used to elaborate in Figure 1 and Supplementary Figure S2 were provided.

## References

[1] Parte A.C., Carbasse J.S., Meier-Kolthoff J.P., Reimer L.C., Göker M. (2020). List of Prokaryotic names with Standing in Nomenclature (LPSN) moves to the DSMZ. Int. J. Syst. Evol. Microbiol..

[2] Lalucat J., Gomila M., Mulet M., Zaruma A., García-Valdés E. (2022). Past, present and future of the boundaries of the *Pseudomonas* genus: Proposal of *Stutzerimonas* gen. nov. Syst. Appl. Microbiol..

[3] Ursing J.B., Rosselló-Mora R.A., Garcia-Valdés E., Lalucat L. (1995). Taxonomic note: A pragmatic approach to the nomenclature of phenotypically similar genomic groups. Int. J. Syst. Evol..

[4] Lalucat J., Bennasar A., Bosch R., García-Valdés E., Palleroni N.J. (2006). Biology of *Pseudomonas stutzeri*. Microbiol. Mol. Biol. Rev..

[5] Oren A., Garrity G.M. (2022). Valid publication of new names and new combinations effectively published outside the IJSEM. Int. J. Syst. Evol. Microbiol..

[6] Parks D.H., Chuvochina M., Waite D.W., Rinke C., Skarshewski A., Chaumeil P.A., Hugenholtz P. (2018). A standardized bacterial taxonomy based on genome phylogeny substantially revises the tree of life. Nat. Biotech..

[7] Richter M., Rosselló-Móra R., Glöckner F.O., Peplies J. (2016). JSpeciesWS: A web server for prokaryotic species circumscription based on pairwise genome comparison. Bioinformatics.

[8] Meier-Kolthoff J.P., Auch A.F., Klenk H.P., Göker M. (2013). Genome sequence-based species delimitation with confidence intervals and improved distance functions. BMC Bioinform..

[9] Meier-Kolthoff J.P., Sardà Carbasse J., Peinado-Olarte R.L., Göker M. (2022). TYGS and LPSN: A database tandem for fast and reliable genome-based classification and nomenclature of prokaryotes. Nucleic Acid Res..

[10] Staley J.T., Pavlinov I. (2013). Transitioning toward a universal species concept for the classification of all organisms. The Species Problem.

[11] Avram O., Rapoport D., Portugez S., Pupko T. (2019). M1CR0B1AL1Z3R—A user-friendly web server for the analysis of large-scale microbial genomics data. Nucleic Acids Res..

[12] Rodriguez-R L.M., Konstantinidis K.T. (2016). The enveomics collection: A toolbox for specialized analyses of microbial genomes and metagenomes. Peer J. Prepr..

[13] Hammer O., Harper D.A.T., Ryan P.D. (2001). Past: Paleontological Statistics Software Package for Education and Data Analysis. Palaeontol. Electron..

[14] Huson D.H., Bryant D. (2006). Application of Phylogenetic Networks in Evolutionary Studies. Mol. Biol. Evol..

[15] Cowan S.T. (1974). Cowan and Steel’s Manual for the Identification of Medical Bacteria.

[16] Lalucat J., Mayer F. (1988). Analysis of Refractile (R) Bodies. Methods in Microbiology.

[17] Rosselló-Mora R.A., Lalucat J., Dott W., Kämpfer P. (1994). Biochemical and chemotaxonomic characterization of *Pseudomonas stutzeri* genomovars. J. Appl. Bacteriol..

[18] Lalucat J., Mulet M., Gomila M., García-Valdés E. (2020). Genomics in bacterial taxonomy: Impact on the genus *Pseudomonas*. Genes.

[19] Li X., Yang Z., Wang W., Li W., Zhang G., Yan H. (2022). Comparative genomics of *Pseudomonas stutzeri* complex: Taxonomic assignments and genetic diversity. Front. Microbiol..

[20] Girard L., Lood C., Höfte M., Vandamme P., Rokni-Zadeh H., van Noort V., Lavigne R., De Mot R. (2021). The ever-expanding *Pseudomonas* genus: Description of 43 new species and partition of the *Pseudomonas putida* group. Microorganisms.

[21] Rosselló R., Garcia-Valdés E., Lalucat J., Ursing J. (1991). Genotypic and phenotypic diversity of *Pseudomonas stutzeri*. Syst. Appl. Microbiol..

[22] Scotta C., Gomila M., Mulet M., Lalucat J., García-Valdés E. (2013). Whole-cell MALDI-TOF mass spectrometry and multilocus sequence analysis in the discrimination of *Pseudomonas stutzeri* populations: Three novel genomovars. Microb. Ecol..

[23] Mulet M., Gomila M., Ramírez A., Lalucat J., Garcia-Valdes E. (2019). *Pseudomonas nosocomialis* sp. nov., isolated from clinical specimens. Int. J. Syst. Evol. Microbiol..

[24] Wolterink A.F.W.M., Jonker A.B., Kengen S.W.M., Stams A.J.M. (2002). *Pseudomonas chloritidismutans* sp. nov., a non-denitrifying, chlorate-reducing bacterium. Int. J. Syst. Evol. Microbiol..

[25] Cladera A.M., García-Valdés E., Lalucat J. (2006). Genotype versus phenotype in the circumscription of bacterial species: The case of *Pseudomonas stutzeri* and *Pseudomonas chloritidismutans*. Arch. Microbiol..

[26] Mulet M., Bennasar A., Lalucat L., García-Valdés E. (2009). An rpoD-based PCR procedure for the identification of *Pseudomonas* species and for their detection in environmental samples. Mol. Cell Probes.

[27] Anwar N., Rozahon M., Zayadan B., Mamtimin H., Abdurahman M., Kurban M., Abdurusul M., Mamtimin T., Abdukerim M., Rahman E. (2017). *Pseudomonas tarimensis* sp. nov., an endophytic bacteria isolated from *Populus euphratica*. Int. J. Syst. Evol. Microbiol..

[28] Peng J.S., Liu Y., Yan L., Hou T.T., Liu H.C., Zhou Y.G., Liu Z.P. (2019). *Pseudomonas nitrititolerans* sp. nov., a nitrite-tolerant denitrifying bacterium isolated from a nitrification/denitrification bioreactor. Int. J. Syst. Evol. Microbiol..

[29] Zou Y., He S., Sun Y., Zhang X., Liu Y., Cheng Q. (2019). *Pseudomonas urumqiensis* sp. nov., isolated from rhizosphere soil of *Alhagi sparsifolia*. Int. J. Syst. Evol. Microbiol..

[30] Baumann P., Bowditch R.D., Baumann L., Beaman B. (1983). Taxonomy of marine *Pseudomonas* species: *P. stanieri* sp. nov.; *P. perfectomarina* sp. nov., nom. rev.; *P. nautica*; and *P. doudoroffii*. Int. J. Syst. Bacteriol..

[31] Zhang L., Pan Y., Wang K., Zhang X., Zhang C., Zhang S., Fu X., Jiang J. (2015). *Pseudomonas zhaodongensis* sp. nov., isolated from saline and alkaline soils. Int. J. Syst. Evol. Microbiol..

[32] Xie F., Ma H., Quan S., Liu D., Chen G., Chao Y., Qian S. (2014). *Pseudomonas kunmingensis* sp. nov., an exopolysaccharide-producing bacterium isolated from a phosphate mine. Int. J. Syst. Evol. Microbiol..

[33] Zobell C.E., Upham H.C. (1944). A list of marine bacteria including description of sixty species. Bull. Scripps Inst. Oceanogr..

[34] Zumft W.G. (1997). Cell biology and molecular basis of denitrification. Microbiol. Mol. Biol. Rev..

[35] Döhler K., Huss V.A.R., Zumft W.G. (1987). Transfer of *Pseudomonas perfectomarina* Baumann, Bowditch, Baumann, and Beaman 1983 to *Pseudomonas stutzeri* (Lehmann and Neumann 1896) Sijderius 1946. Int. J. Syst. Evol. Microbiol..

[36] Rosselló-Mora R.A., García-Valdés E., Lalucat J. (1993). Taxonomic relationship between *Pseudomonas perfectomarina* ZoBell and *Pseudomonas stutzeri*. Int. J. Syst. Evol. Microbiol..

[37] Peña A., Busquets A., Gomila M., Bosch R., Nogales B., García-Valdés E., Lalucat J., Bennasar A. (2012). Draft genome of *Pseudomonas stutzeri* strain ZoBell (CCUG 16156), a marine isolate and model organism for denitrification studies. J. Bacteriol..

[38] Stanier R.Y., Palleroni N.J., Doudoroff M. (1966). The aerobic *Pseudomonas*: A taxonomic study. J. Gen. Microbiol..

[39] Mehboob F., Junca H., Schraa G., Stams A.J. (2009). Growth of *Pseudomonas chloritidismutans* AW-1(T) on n-alkanes with chlorate as electron acceptor. Appl. Microbiol. Biotechnol..

[40] Youngblut M.D., Wang O., Barnum T.P., Coates J.D. (2016). (Per)chlorate in Biology on Earth and Beyond. Annu. Rev. Microbiol..

[41] Rosselló-Mora R.A. (1992). Caracterización Taxonómica de Cepas de *Pseudomonas stutzeri* Degradadoras y no Degradadoras de Naftaleno. Ph.D. Thesis.

