# Supplementary material for: Genome-Based Taxonomy of the Genus Stutzerimonas and Proposal of S. frequens sp. nov. and S. degradans sp. nov. and Emended Descriptions of S. perfectomarina and S. chloritidismutans
Source: Microorganisms. 2022 Jul 6;10(7):1363. doi: 10.3390/microorganisms10071363 (PMC9320692; doi:10.3390/microorganisms10071363)
Supplement: Supplementary file 1 [file microorganisms-10-01363-s001.zip › microorganisms-1757643-supplementary/Supplementary materials_Figures_DEF.pdf]

Supplementary materials

Genome-based taxonomy of the genus *Stutzerimonas* and proposal of *S. frequens* sp. nov. and *S. degradans* sp. nov. and emended descriptions of *S. perfectomarina* and *S. chloritidismutans*

Margarita Gomila [1], Magdalena Mulet [2], Elena García-Valdés [1,2] and Jorge Lalucat[1]

1 Universitat de les Illes Balears; Palma de Mallorca (Spain)

2 Institut Mediterrani d'Estudis Avançats (IMEDEA CSIC-UIB)

* Correspondence: jlalucat@uib.es

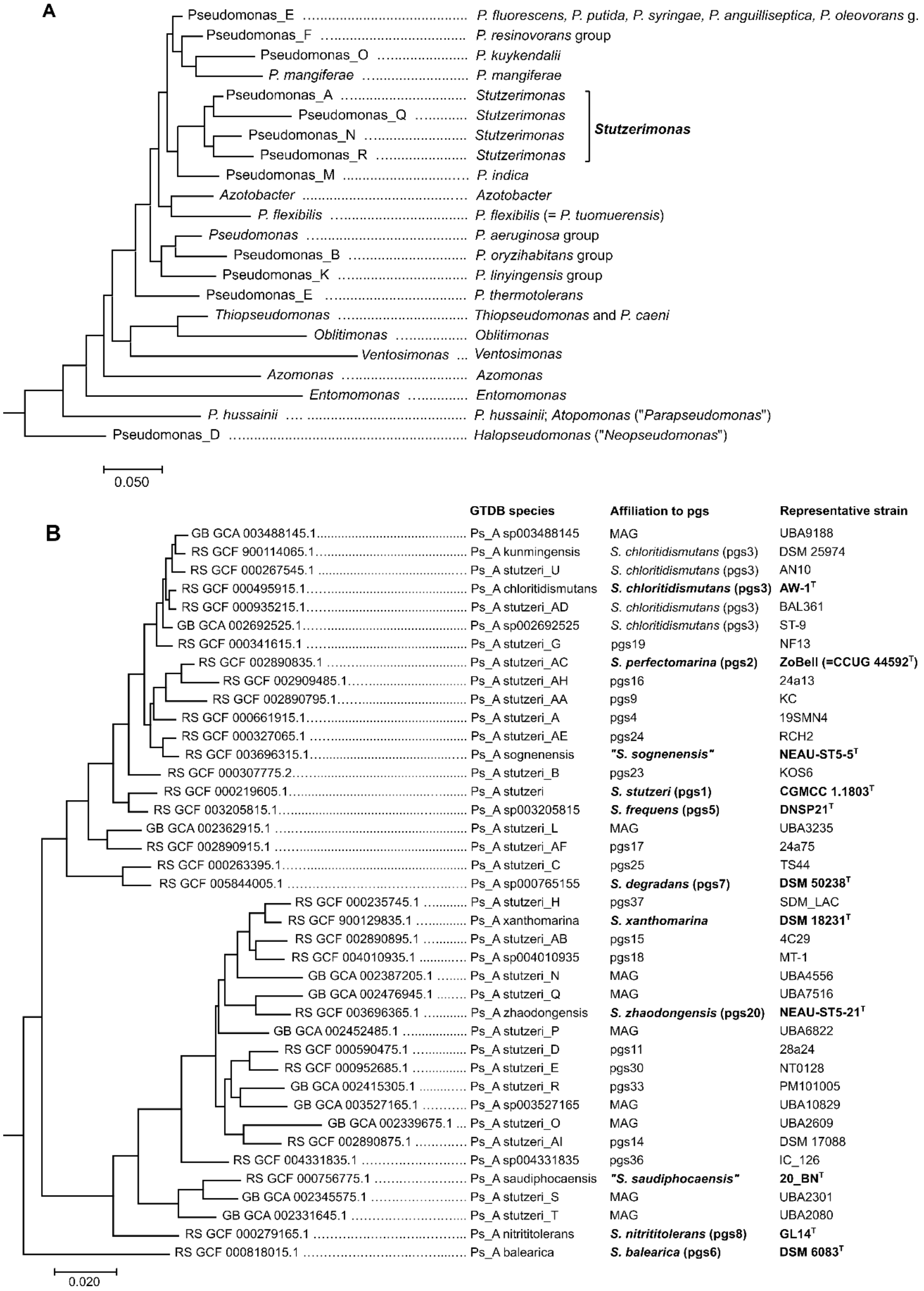

Suppl Figure S1. Phylogenetic analysis obtained at the GTDB-Anno tree web site of representative strains in the *Pseudomonadaceae* (A) and in *Stutzerimonas* (B).

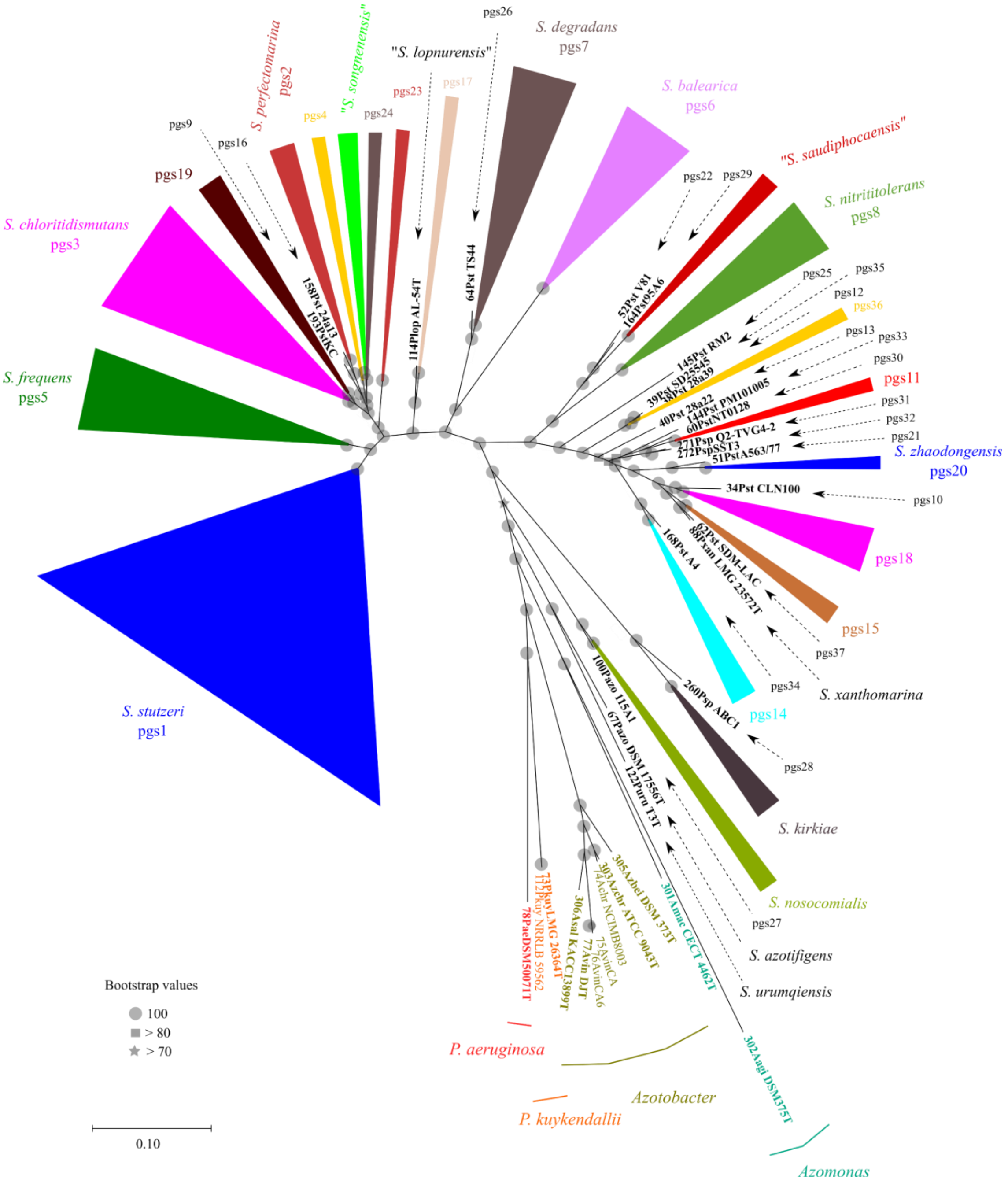

Suppl Figure S2. Core-genome phylogeny based on the concatenated sequences of 666 core genes of 200 genomes including members of *Azotobacter*, *Azomonas* and *Pseudomonas* species as outgroup. Tree was constructed using the maximum likelihood method. Bootstrap values of 100 rep-licates are indicated in the nodes with different symbols: circles indicate bootstrap values of 100, squares indicate values higher than 80% and stars indicate values higher than 70%.

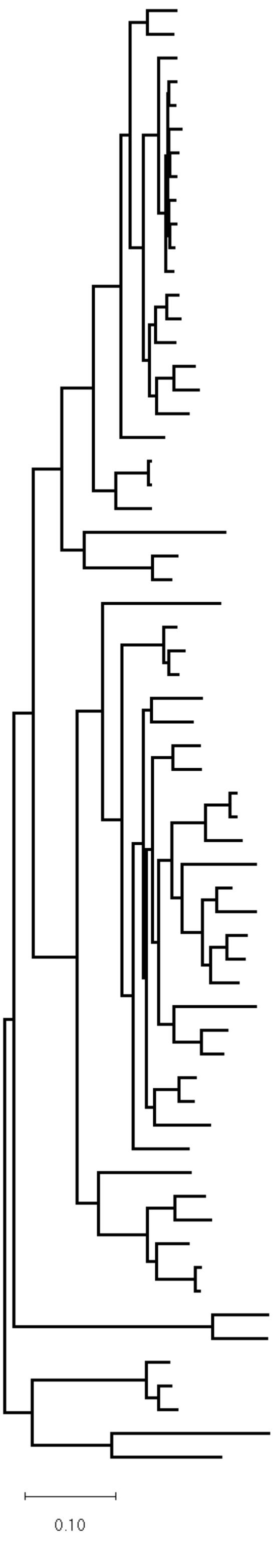

Genome nr	Core-genome phylogeny			GTDB Taxonomy		
	Proposed or accepted name	pgs	Reference strain	Proposed name	nr of genomes in species cluster	MAGs in species cluster
185	***S. frequens***	pgs5	DNSP21	Ps_A sp003205815	20	12
1	***S. stutzeri***	pgs1	ATCC 17588	Ps_A stutzeri	147	95
48	*Stutzerimonas* sp.	pgs19	NF13	Ps_A stutzeri_G	3	2
124	*S. chloritidismutans*	pgs3	PX_371	Ps_A xanthomarina_A	17	15
17	*S. chloritidismutans*	pgs3	ST-9	Ps_A sp002692525	7	6
sp003488145	*S. chloritidismutans*	pgs3	UBA9188	Ps_A sp003488145	2	2
19	*S. chloritidismutans*	pgs3	CCUG 29243	Ps_A stutzeri_U	2	0
		pgs3	CAL	Ps_A sp007713455	1	1
111	*S. chloritidismutans*	pgs3	DSM 25974 T	Ps_A kunmingensis	3	2
23	***S. chloritidismutans***	pgs3	AW-1 T	Ps_A chloritidismutans	7	4
	S. chloritidismutans	pgs3	S11	Ps_A kunmingensis_A	10	7
22	*S. chloritidismutans*	pgs3	BAL361	Ps_A stutzeri_AD	3	2
65	*Stutzerimonas* sp.	pgs24	RCH2	Ps_A stutzeri_AE	3	1
71	***"S. songnenensis"***		DSM 27560	Ps_A songnenensis	4	1
24	*Stutzerimonas* sp.	pgs4	19SMN4	Ps_A stutzeri_A	2	0
175	***S. perfectomarina***	pgs2	CCUG 44592	Ps_A stutzeri_AC	13	9
158	*Stutzerimonas* sp.	pgs16	24a13	Ps_A stutzeri_AH	1	0
193	*Stutzerimonas* sp.	pgs9	KC	Ps_A stutzeri_AA	1	0
58	*Stutzerimonas* sp.	pgs23	KOS6	Ps_A stutzeri_B	1	1
PsAstL			UBA3235	Ps_A stutzeri_L	3	3
114	***"S. lopnurensis"***		AL-54			
159	*Stutzerimonas* sp.	pgs17	24a75	Ps_A stutzeri_AF	3	1
68	***S. balearica***	pgs6	DSM6083	Ps_A balearica	10	5
64	*Stutzerimonas* sp.	pgs26	TS44	Ps_A stutzeri_C	1	0
189	***S. degradans***	pgs7	DSM 50238	Ps_A sp000765155	9	0
145	*Stutzerimonas* sp.	pgs25	RM2			
165	*Stutzerimonas* sp.	pgs36	99A1	Ps_A sp004331835	2	2
39	*Stutzerimonas* sp.	pgs35	SD25545			
38	*Stutzerimonas* sp.	pgs12	28a39	Ps_A stutzeri_AI	4	1
144	*Stutzerimonas* sp.	pgs33	PM101005	Ps_A stutzeri_R	2	1
sp003527165			UBA10829	Ps_A sp003527165	1	1
60	*Stutzerimonas* sp.	pgs30	NT0128	Ps_A stutzeri_E	1	0
36	*Stutzerimonas* sp.	pgs11	28a50	Ps_A stutzeri_D		0
51	*Stutzerimonas* sp.	pgs21	77/147			
PsAstQ		pgs21	UBA7516	Ps_A stutzeri_Q	1	1
50	***S. zhaodongensis***	pgs20	NEAU-ST5-21 T	Ps_A zhaodongensis	1	0
PsAstN			UBA4556	Ps_A stutzeri_N	2	2
45	*Stutzerimonas* sp.	pgs18	MT1	Ps_A sp004010935	11	4
34	*Stutzerimonas* sp.	pgs10	CLN100			
62	*Stutzerimonas* sp.	pgs37	SDM-LAC	Ps_A stutzeri_H	1	0
88	***S. xanthomarina***		LMG 23572 T	Ps_A xanthomarina	2	0
155	*Stutzerimonas* sp.	pgs15	4C29	Ps_A stutzeri_AB	11	7
PsAstO			UBA2609	Ps_A stutzeri_O	1	1
168	*Stutzerimonas* sp.	pgs34	A4			
161	*Stutzerimonas* sp.	pgs14	28a39			
272	*Stutzerimonas* sp.	pgs31	SST3	Ps_A sp003325755	1	0
271	*Stutzerimonas* sp.	pgs32	Q2-TVG4-2	Ps_A sp013620795	1	0
PsAstP			UBA6822	Ps_A stutzeri_P	1	1
40	*Stutzerimonas* sp.	pgs13	28a22			0
115	***S. nitrititolerans***	pgs8	GL14 T	Ps_A nitrititolerans	51	41
PsAstS			UBA2301	Ps_A stutzeri_S	1	1
246	***"S. suadiphocaensis"***		20_BN T	Ps_A saudiphocaensis	2	2
164	*Stutzerimonas* sp.	pgs29	95A6	Ps_A stutzeri_AK	1	1
PsAstT		pgs22	UBA2080	Ps_A stutzeri_T	1	1
52	*Stutzerimonas* sp.	pgs22	V81	Ps_A stutzeri_T		
260	*Stutzerimonas* sp.	pgs28	ABC1	Pseudomonas_Q sp013395055	1	0
300	***S. kirkiae***		P4C T	Pseudomonas_Q kirkiae	4	0
100	*Stutzerimonas* sp.	pgs27	115A1	Pseudomonas_R azotifigens_A	1	0
54	***S. nosocomialis***		A31/70 T	Pseudomonas_R nosocomialis	3	0
			HGM22033	Ps_R sp900766265	1	1
122	***S. urumqiensis***		T3 T	Pseudomonas_N sp003640395	1	0
67	***S. azotifigens***		DSM 17556 T	Pseudomonas_N azotifigens	1	0

Supp Figure S3. Correspondence of the core-genome phylogeny and the GTDB taxonomy among 62 genomes of pgs representative strains and GTDB representative genomes. The length of the concatenated sequences of the 758 core genes is 284,095 nucleotides. Species names in the current taxonomy are labelled in bold. The ML tree was rooted on midpoint. Bar indicates sequence divergence.

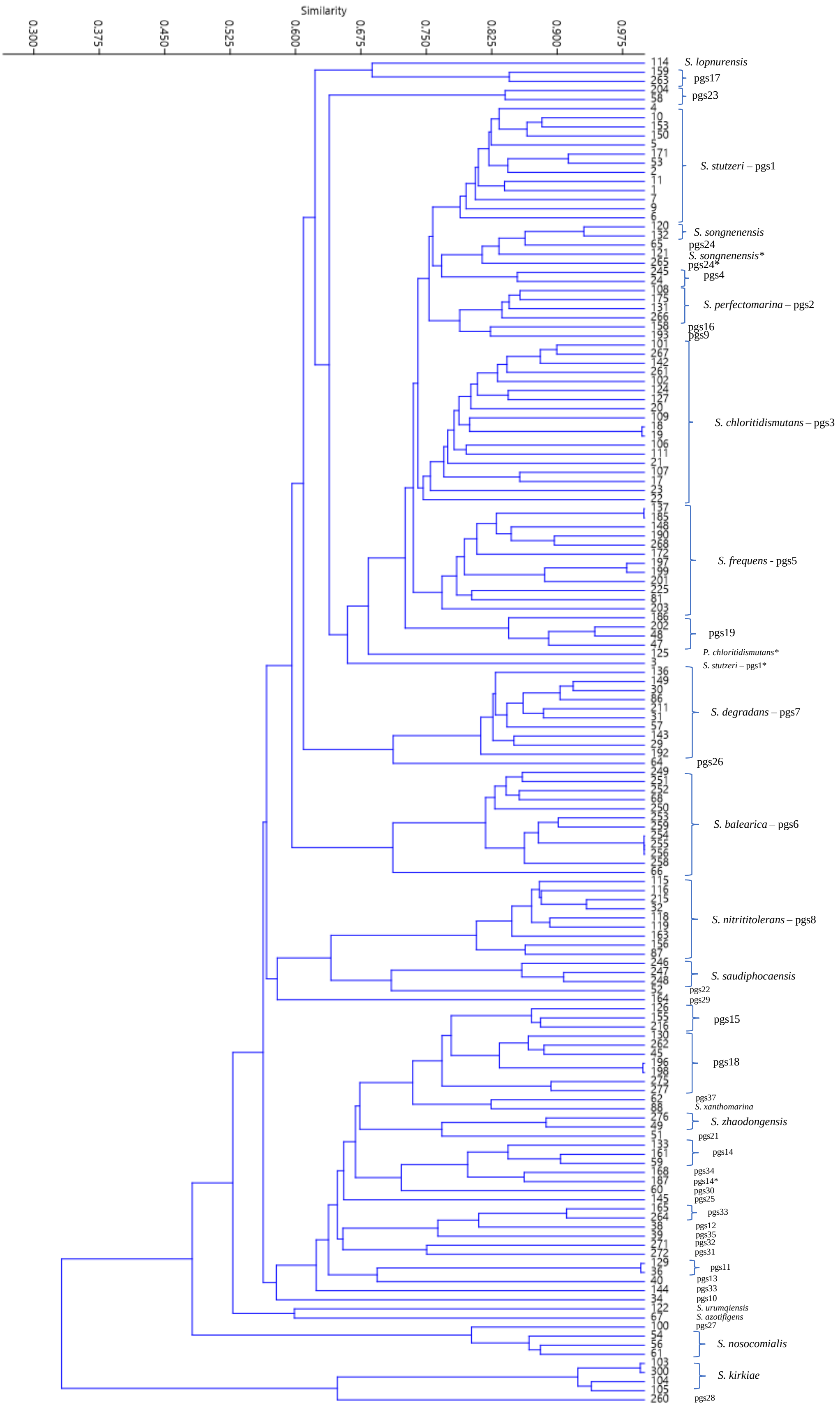

Suppl Figure S4. UPGMA dendrogram based on the Jaccard index calculated from the phyletic pattern. * indicates the exceptions in the grouping pattern.

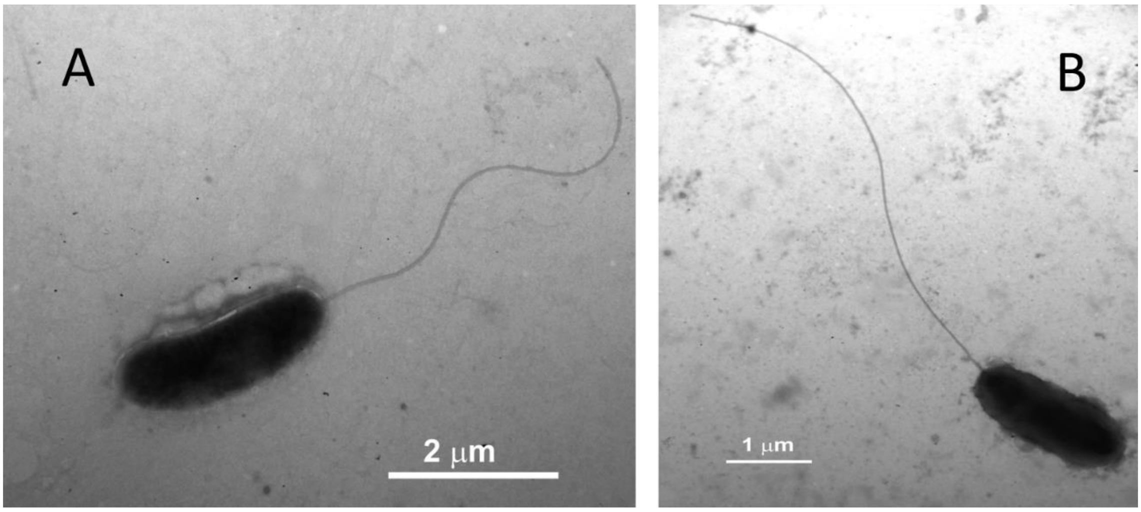

Suppl Figure S5. Transmission electron micrograph of negatively stained cells of *S. frequens* strain DNSP21T (A) and *S. degradans* strain DSM 50238T (B) showing the polar inserted flagellum.
